# Biallelic and monoallelic variants in *PLXNA1* are implicated in a novel neurodevelopmental disorder with variable cerebral and eye anomalies

**DOI:** 10.1038/s41436-021-01196-9

**Published:** 2021-05-30

**Authors:** Gabriel C. Dworschak, Jaya Punetha, Jeshurun C. Kalanithy, Enrico Mingardo, Haktan B. Erdem, Zeynep C. Akdemir, Ender Karaca, Tadahiro Mitani, Dana Marafi, Jawid M. Fatih, Shalini N. Jhangiani, Jill V. Hunter, Tikam Chand Dakal, Bhanupriya Dhabhai, Omar Dabbagh, Hessa S. Alsaif, Fowzan S. Alkuraya, Reza Maroofian, Henry Houlden, Stephanie Efthymiou, Natalia Dominik, Vincenzo Salpietro, Tipu Sultan, Shahzad Haider, Farah Bibi, Holger Thiele, Julia Hoefele, Korbinian M. Riedhammer, Matias Wagner, Ilaria Guella, Michelle Demos, Boris Keren, Julien Buratti, Perrine Charles, Caroline Nava, Delphine Héron, Solveig Heide, Elise Valkanas, Leigh B. Waddell, Kristi J. Jones, Emily C. Oates, Sandra T. Cooper, Daniel MacArthur, Steffen Syrbe, Andreas Ziegler, Konrad Platzer, Volkan Okur, Wendy K. Chung, Sarah A. O’Shea, Roy Alcalay, Stanley Fahn, Paul R. Mark, Renzo Guerrini, Annalisa Vetro, Beth Hudson, Rhonda E. Schnur, George E. Hoganson, Jennifer E. Burton, Meriel McEntagart, Tobias Lindenberg, Öznur Yilmaz, Benjamin Odermatt, Davut Pehlivan, Jennifer E. Posey, James R. Lupski, Heiko Reutter

**Affiliations:** 1 Institute of Human Genetics, Medical Faculty, University of Bonn, Bonn, Germany; 2 Institute of Anatomy and Cell Biology, Medical Faculty, University of Bonn, Bonn, Germany; 3 Department of Pediatrics, University Hospital Bonn, Bonn, Germany; 4 Department of Molecular and Human Genetics, Baylor College of Medicine, Houston, TX USA; 5 Department of Genetics and Genomic Sciences, Icahn School of Medicine at Mount Sinai, New York, NY USA; 6 Department of Medical Genetics, University of Health Sciences, Diskapi Yildirim Beyazit Training and Research Hospital, Ankara, Turkey; 7 Department of Genetics, University of Alabama at Birmingham, Birmingham, AL USA; 8 Human Genome Sequencing Center, Baylor College of Medicine, Houston, TX USA; 9 Department of Radiology, Baylor College of Medicine, Houston, TX USA; 10 Genome and Computational Biology Lab, Department of Biotechnology, Mohanlal Sukhadia University, Udaipur, Rajasthan India; 11 Department of Neuroscience, King Faisal Specialist Hospital and Research Center, Riyadh, Saudi Arabia; 12 Department of Genetics, King Faisal Specialist Hospital and Research Centre, Riyadh, Saudi Arabia; 13 Department of Anatomy and Cell Biology, College of Medicine, Alfaisal University, Riyadh, Saudi Arabia; 14 Department of Neuromuscular Disorders, UCL Queen Square Institute of Neurology, University College London, London, United Kingdom; 15 Department of Pediatric Neurology, Institute of Child Health, The Children’s Hospital Lahore, Lahore, Pakistan; 16 Department of Paediatric Medicine, Wah Medical College, Rawalpindi, Pakistan; 17 University Institute of Biochemistry & Biotechnology, PMAS – Arid Agriculture University, Rawalpindi, Pakistan; 18 Cologne Center for Genomics, University of Cologne, Cologne, Germany; 19 Institute of Human Genetics, Klinikum rechts der Isar, School of Medicine, Technical University of Munich, Munich, Germany; 20 Department of Nephrology, Klinikum rechts der Isar, School of Medicine, Technical University of Munich, Munich, Germany; 21 Institute of Human Genetics, Helmholtz Zentrum München, Neuherberg, Germany; 22 Institute of Neurogenomics, Helmholtz Zentrum München, Neuherberg, Germany; 23 Department of Medical Genetics, Centre for Applied Neurogenetics, University of British Columbia, Vancouver, BC Canada; 24 Division of Neurology, Department of Pediatrics, University of British Columbia and BC Children’s Hospital, Vancouver, BC Canada; 25 AP-HP, Hôpital Pitié-Salpêtrière, Département de Génétique, Paris, France; 26 Institut du Cerveau et de la Moelle épinière, Sorbonne Université, UMR S 1127, Inserm U1127, CNRS UMR 7225, Paris, France; 27 Center for Mendelian Genomics, The Broad Institute of Massachusetts Institute of Technology and Harvard, Cambridge, MA USA; 28 Kids Neuroscience Centre, Kids Research, The Children’s Hospital at Westmead, Sydney, NSW Australia; 29 Discipline of Child and Adolescent Health, Sydney Medical School, University of Sydney, Sydney, NSW Australia; 30 School of Biotechnology and Biomolecular Sciences, Faculty of Science, The University of New South Wales, Sydney, NSW Australia; 31 Children’s Medical Research Institute, Westmead, NSW Australia; 32 Broad Institute of Harvard and Massachusetts Institute of Technology, Cambridge, MA USA; 33 Analytic and Translational Genetics Unit, Massachusetts General Hospital, Boston, MA USA; 34 Harvard Medical School, Boston, MA USA; 35 Division of Pediatric Epileptology, Centre for Paediatrics and Adolescent Medicine, University Hospital Heidelberg, Heidelberg, Germany; 36 Division of Pediatric Neurology and Metabolic Medicine, Centre for Pediatrics and Adolescent Medicine, University Hospital Heidelberg, Heidelberg, Germany; 37 Institute of Human Genetics, University of Leipzig Medical Center, Leipzig, Germany; 38 Department of Pediatrics, Columbia University, New York, NY USA; 39 Department of Neurology, Columbia University, New York, NY USA; 40 Division of Medical Genetics, Helen DeVos Children’s Hospital Grand Rapids, New York, MI USA; 41 Neuroscience Department, Children’s Hospital A. Meyer-University of Florence, Florence, Italy; 42 GeneDx, Gaithersburg, MD USA; 43 Department of Pediatrics, University of Illinois, College of Medicine, Chicago, IL USA; 44 Department of Pediatrics, University of Illinois, College of Medicine, Peoria, IL USA; 45 South West Thames Regional Genetics Centre, St. George’s Healthcare NHS Trust, St. George’s, University of London, London, United Kingdom; 46 Institute of Neuroanatomy, Medical Faculty, University of Bonn, Bonn, Germany; 47 Section of Neurology, Department of Pediatrics, Baylor College of Medicine, Houston, TX USA; 48 Texas Children’s Hospital, Houston, TX USA; 49 Department of Pediatrics, Baylor College of Medicine, Houston, TX USA; 50 Department of Neonatology and Pediatric Intensive Care, University Hospital Bonn, Bonn, Germany

## Abstract

**Purpose:**

To investigate the effect of *PLXNA1* variants on the phenotype of patients with autosomal dominant and recessive inheritance patterns and to functionally characterize the zebrafish homologs *plxna1a* and *plxna1b* during development.

**Methods:**

We assembled ten patients from seven families with biallelic or de novo *PLXNA1* variants. We describe genotype–phenotype correlations, investigated the variants by structural modeling, and used Morpholino knockdown experiments in zebrafish to characterize the embryonic role of *plxna1a* and *plxna1b*.

**Results:**

Shared phenotypic features among patients include global developmental delay (9/10), brain anomalies (6/10), and eye anomalies (7/10). Notably, seizures were predominantly reported in patients with monoallelic variants. Structural modeling of missense variants in *PLXNA1* suggests distortion in the native protein. Our zebrafish studies enforce an embryonic role of *plxna1a* and *plxna1b* in the development of the central nervous system and the eye.

**Conclusion:**

We propose that different biallelic and monoallelic variants in *PLXNA1* result in a novel neurodevelopmental syndrome mainly comprising developmental delay, brain, and eye anomalies. We hypothesize that biallelic variants in the extracellular Plexin-A1 domains lead to impaired dimerization or lack of receptor molecules, whereas monoallelic variants in the intracellular Plexin-A1 domains might impair downstream signaling through a dominant-negative effect.

## INTRODUCTION

Plexins are a large family of cell surface receptors for the axon guidance molecules semaphorins. Plexin-A1 and its co-receptor Neuropilin-1 (NRP1) bind different classes of semaphorins.^1–4^ The Plexin cytoplasmic domain contains two segments (C1 and C2) that have sequence similarity to GTPase-activating protein (GAP) and form a functional GAP domain.^5^ Before semaphorin binding, plexin is an inactive monomer or dimer in which the RapGAP activity is autoinhibited.^6^ Semaphorin-induced dimerization of the plexin extracellular region promotes formation of the activating dimer of the cytoplasmic region, which converts the GAP domain to the active state through an allosteric mechanism.^6–8^ The Plexin-A1 GAP domains show dual specificity for Rac and Rap GTPases.^9^ *Plxna1* null mice exhibit different axonal abnormalities (e.g., abnormal proprioceptive neuronal and oligodendrocyte morphology, slight defasciculation of optic chiasm, aberrant crossing of commissural axons, agenesis of corpus callosum [CC], and defects in the olfactory and neuroendocrine reproductive systems).^10–13^ Additionally, *Plxna1* null mice exhibit neuronal abnormalities with rarefied interneurons in developing cortex and a decreased cortical thickness.^14^ Recently, van der Klaauw et al. implicated rare monoallelic variants in plexins and semaphorins in the expression of severe obesity.^15^ They found 40 rare variants in 13 plexin and semaphorin genes. Notably, nine variants were associated with neurodevelopmental phenotypes in the respective patients. Previously, three studies reported monoallelic de novo variants in *PLXNA1* to be associated with infantile-onset epilepsy, intellectual disability with autism spectrum disorder (ASD), epileptic encephalopathy, or schizophrenia in the respective patients.^16–19^

Here, we describe four families with rare biallelic and three families with rare/novel monoallelic de novo variants in *PLXNA1*. The observed clinical phenotypes establish a range of neurological disease associated with presumably pathogenic variant alleles at this locus. Shared phenotypic features comprise global developmental delay, brain and eye anomalies. Seizures were predominantly reported in patients with monoallelic variants. Morpholino knockdown of the zebrafish homologs *plxna1a* and *plxna1b* in zebrafish larvae causes anomalies of the central nervous system and the eye as observed in our patients.

## MATERIALS AND METHODS

### Exome sequencing

Exome sequencing and subsequent analysis was performed by established procedures (see Supplementary information). GeneMatcher^20,21^ and matchbox^22^ facilitated the identification of additional patients with biallelic and monoallelic pathogenic variants in *PLXNA1*.

### 3D modeling of protein structure

The 3D protein structural models were built using I-Tasser.^23^ Sequences were trimmed from the N-terminal (1,020 amino acids) for prediction of the respective Plexin-A1 amino acid changes. Structural comparison of variants were done in Chimera after superimposing the structure of mutant onto the wild-type structure using SuperPose (superpose.wishartlab.com). Amino acid conservation was obtained from the Consurf server.^24^

### Zebrafish husbandry and embryo maintenance

Zebrafish were maintained and raised according to national law and recommendations by Westerfield^25^ in our fish facility in Bonn, Germany. Zebrafish larvae (zfl) of wild-type AB/TL strain and transgenic *Tg*(*-3.1ngn1:GFP*)^26^ were obtained by natural spawning and raised at 28 °C on a 14-hour light–10-hour dark cycle.

### Knockdown with morpholino oligonucleotides microinjections and mRNA rescue

The human *PLXNA1* gene has two zebrafish orthologs (*plxna1a*, ENSDARG00000105452; and *plxna1b*, ENSDARG00000114823).^27^ Knockdown was performed using specific Morpholino® oligonucleotides (MO) synthetized by GeneTools, LLC. We designed one MO targeting a splice site (splice blocking [SB]) and one MO targeting the AUG translational start site (translational blocking [TB]) for each of the orthologs. In one-cell or two-cell embryos 2.2 ng (1.7 nL/embryo) of *plxna1a* SB MO (5’-AAGGAGATGCAGATACTTACACACT-’3), 2.9 ng *plxna1a* TB MO (5’-CCCCTACCATACGGCAGCATTTTTC-’3), 4.4 ng *plxna1b* SB MO (5’-AGCAGATAATTCTCTTACCGAGATC-’3), 1.5 ng *plxna1b* TB MO (5’-GCCACATATCTGCACTGGTCCTTGA-’3), or 4.4 ng of standard control MO (5’-CCTCTTACCTCAGTTACAATTTATA-‘3) was injected into the yolk. *plxna1b* SB MO and *plxna1b* TB MO were established previously in a model for wound healing but not early embryonic development.^28,29^

For messenger RNA (mRNA) rescue experiments, 150 pg of in vitro transcribed human *PLXNA1* mRNA was co-injected into the yolk of one-cell embryos together with *plxna1a* SB MO. *PLXNA1* mRNA was transcribed from complementary DNA (cDNA) clone HsCD00863277 (Harvard Medical School) containing NM_032242.3 using the mMESSAGE mMACHINE T7 Ultra Kit (Thermo Fisher Scientific). Prior to transcription, the orf of clone HsCD00863277 was changed into the stop codon of NM_032242.3 using the mutagenesis In-Fusion HD Cloning kit (Takara).

### RNA isolation and reverse transcription polymerase chain reaction

To test splice-blocking effect of the designed *plxna1a* SB MO, total RNA was extracted from pools of 20 larvae with TRIzol reagent (Thermo Fisher Scientific). Then, 1 µg of RNA was used for cDNA synthesis with iScript™ Reverse Transcription Supermix (Bio-Rad). Polymerase chain reaction (PCR) was performed with *plxna1a* forward primer (5’- GATGAAGAAGATCTTGGTGAACT-‘3) and intron-spanning *plxna1a* reverse primer (5’- AAGAACCAGCTGGACTTCAG-‘3); for control *eef1α1* was used as housekeeping gene.^30^

### Imaging and phenotyping

Zfl were phenotyped at 2 days postfertilization (dpf) using a ZEISS Stemi508 for brightfield imaging. The timepoint of 2 dpf was chosen since the phenotype was most prominent. The phenotype category is defined by the presence of at least two of the following features: hydrocephalus, general hypopigmentation, reduced head or eye size. Diameter of head and eyes was measured with NIS-Element Viewer software. To account for variation and growth effects, eye size was calculated as diameter normalized to head.^31^ Zfl were anesthetized at 2 dpf with 0.03% tricaine (Sigma-Aldrich), fixed in 1.25% low-melting agarose for fluorescence imaging with a ZEISS Axio Zoom.V16 stereo microscope. Phenotypic differences and dorsal root ganglions (DRG) in *Tg*(*-3.1ngn1:GFP*) were analyzed with the ZEN 2.3 software. To account for variation in embryo size, DRG were counted in somites cranial of the yolk sac and thereby normalized.

### Statistical analyses

Two-tailed Student’s *t*-test, Mantel–Cox, and two-way analysis of variance (ANOVA) were used for analysis using GraphPad Prism version 6. Survival was analyzed using Kaplan–Meier survival curves.

## RESULTS

### Biallelic and monoallelic *PLXNA1* variants

**Fig. 1:**
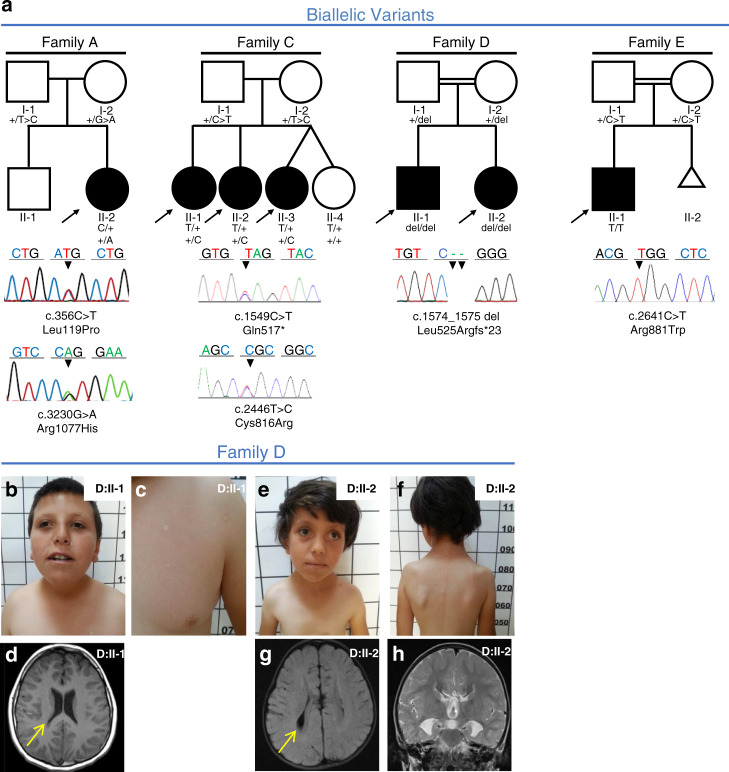
Families with biallelic *PLXNA1* variants. **a** Pedigrees and Sanger sequencing results of four families with biallelic variants in *PLXNA1*. **b**–**h** Photographs and brain magnetic resonance image (MRI) of affected siblings of family D. **b**, **c** D:II-1 showing mild microphthalmia, depressed nasal bridge, short neck, and hypopigmented stains that were absent in both parents. **d** T1 axial MRI of D:II-1 showing dysmorphic ventricular system most prominent in posterior horns (arrow). **e**, **f** D:II-2 showing strabismus, sparse lateral eyebrows, flattened nasal bridge, large earlobes, and hypopigmented lesions on the torso measuring less than 1 cm. **g**, **h** Axial T2 FLAIR MRI of D:II-2 showing dysmorphic ventricular system (arrow in **g**), and frontal steer horn sign typically seen in corpus callosum (CC) agenesis in the T2 coronal (**h**).

In four families, we identified seven patients with biallelic variants in *PLXNA1* segregating with the disease (Fig. 1a). Clinical findings are summarized in Table 1; detailed case reports can be found in the Supplementary information. Six patients showed global developmental delay (6/7) whereas one patient had isolated language regression (1/7). Three patients had cerebral anomalies (3/7). Brain magnetic resonance image (MRI) studies of patient D:II-1 showed dysmorphic ventricular system and prominent Virchow–Robins (perivascular) spaces at the level of the semiovale in both hemispheres. MRI studies of his sister (D:II-2) showed agenesis of the CC and colpocephaly (Fig. 1d, g, h). While the affected patients II-1 and II-3 in family C had unremarkable cerebral MRI studies, their affected sister (C:II-2) showed a dysplastic “mega CC.” Three patients had ASD (3/7), and four had eye anomalies (4/7) comprising optic disc hypoplasia without visual deficits, strabismus, and ptosis in (D:II-1, D:II-2); ptosis in (C:II-2); and nystagmus in (C:II-3). Three patients showed craniofacial dysmorphisms (3/7). Only one patient presented with seizures (1/7); patient A:II-1 had 15 episodes of febrile and nonfebrile seizures between 15 months and 4.5 years of age.

**Table 1:** Clinical features of ten patients with rare and novel biallelic and monoallelic variants in *PLXNA1*.

Family ID	Family A	Family C			Family D		Family E	Family F	Family G	Family H
Patient	II-2	II-1	II-2	II-3	II-1	II-2	II-1	II-1	II-1	II-1
Zygosity	Compound heterozygous	Compound heterozygous			Homozygous		Homozygous	De novo	De novo	De novo
	c.356T>C, p.(Leu119Pro) c.3230G>A, p.(Arg1077His)	c.1549C>T, p.(Gln517*) c.2446T>C, p.(Cys816Arg)			c.1574_1575del, p.(Leu525Argfs*23)		c.2641C>T, p.(Arg881Trp)	c.3554G>A, p.(Arg1185Gln)	c.4483C>T, p.(Arg1495Trp)	c.5242C>T, p.(Arg1748Cys)
gnomAD MAF%	NR; 0.003% (hom=0)	0.001% (hom=0); NR			NR		0.0004% (hom=0)	0.001% (hom=0)	NR	NR
GDD	Yes	Yes	Yes	Yes	Yes	Yes	No^a^	Yes	Yes	Yes
Seizures	15 episodes of febrile and nonfebrile	-	-	-	-	-	-	Neonatal-onset absence-like	Generalized tonic–clonic	Neonatal-onset atonic
Craniofacial anomalies	-	-	Cupped ears, small teeth, microcephaly	-	Sparse lateral eyebrows, depressed nasal bridge, large earlobes, short neck		-	High forehead, hypertelorism, posteriorly rotated ears, smooth philtrum	Unilateral facial palsy, dysmorphic right auricle	Macrocephaly, mild face hypotrophy
Eye anomalies	-	-	Ptosis	Nystagmus	Optic disc hypoplasia, strabismus, mild ptosis		-	Mild ptosis	Optic disc hypoplasia, impaired vision	Enophthalmia
Cerebral anomalies	-	-	Dysplastic “mega” CC	-	Dysmorphic VS, prominent Virchow–Robins spaces	Agenesis of CC, colpocephaly	Several bilateral high signal foci in the subcortical white matter	Normal brain MRI	Enlarged VS, thin CC, brainstem hypoplasia, agenesis of the posterior pituitary	Periventricular leukoencephalopathy, basal ganglia calcifications, subtentorial atrophy
Neurologic findings	-	-	Unitateral sensorineural hearing loss, ASD	-	ADHD, ASD	Bilateral peripheral axonal neuropathy	Lower limb hyper-reflexia, ASD	Muscular hypotonia	Bilateral sensorineural hearing loss, vestibule-cochlear nerve agenesis, muscular hypotonia	Spastic paraparesis, pyramidal signs
Other	Hypoplastic right kidney	VUR		-	Hypopigmented skin lesions		Elevated CK, hypo- and hyperpigmented skin	IUGR, DCM, joint hypermobility, hand bone and rib anomalies	Dextrocardia, SVC, esophageal atresia	-

*ADHD* attention deficit hyperactivity disorder, *ASD* autism spectrum disorder, *CC* corpus callosum, *CK* creatine kinase, *CSF* cerebrospinal fluid, *DCM* dilated cardiomyopathy, *GDD* global developmental delay, *IUGR* intrauterine growth restriction, *MAF* minor allele frequency, *MRI* magnetic resonance image, *NR* not reported, *SVC* left-sided superior vena cava, *VS* ventricular system, *VUR* vesicoureteral reflux.

^a^Isolated language regression.

**Fig. 2:**
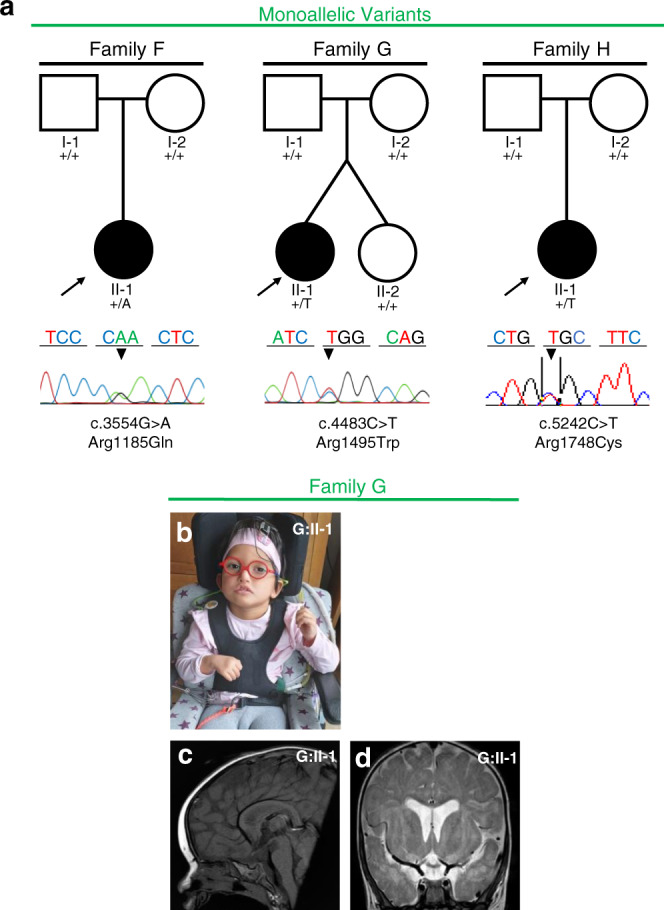
Families with monoallelic *PLXNA1* variants. **a** Pedigrees and Sanger sequencing results of three families with monoallelic de novo variants in *PLXNA1*. **b**–**d** Photographs and brain magnetic resonance image (MRI) of affected patient of family G. **b** G:II-1 showing unilateral facial palsy, dysmorphic right auricle and bilateral sensorineural hearing loss due to agenesis of vestibulocochlear nerves requiring cochlear implants. **c** Midsagittal T1 MRI of G:II-1 showing absent posterior pituitary and mild hypoplasia of brainstem. **d** Coronal T2 MRI of G:II-1 showing mild dilatation of the ventricular system, delayed myelination including the periventricular region.

In three further patients, we identified monoallelic de novo variants in *PLXNA1* (Fig. 2a). Clinical findings are summarized in Table 1; detailed case reports can be found in the Supplementary information. All three patients showed global developmental delay (3/3) and craniofacial dysmorphisms (3/3). Two had hypotonia (2/3) and two had cerebral anomalies (2/3). MRI studies of patient H:II-1 showed periventricular leukoencephalopathy, basal ganglia calcifications, and infratentorial atrophy. MRI studies of patient G:II-1 showed enlarged ventricular system, mild thinning of the CC, delayed myelination, hypoplasia of the brainstem, and agenesis of the posterior pituitary (Fig. 2c, d). All three had eye anomalies (3/3) characterized by enophthalmia (H:II-1), optic disc hypoplasia with impaired vision (G:II-1), and ptosis (F:II-1). Notably, all three patients presented with seizures (3/3) including neonatal-onset atonic seizures (H:II-1), childhood-onset generalized tonic–clonic seizures (G:II-1), and neonatal-onset absence-like seizures (F:II-1).

Besides the above reported patients, we identified four additional patients with biallelic and five additional patients with monoallelic variants in *PLXNA1*. In all patients, the clinical significance of the identified variants remains uncertain. In one of the patients with biallelic variants (family J) and in three of the patients with monoallelic variants (families M, N, and P), either no parent or only one parent was available for segregation analysis. Patient L:II-3, carrying a rare de novo variant, showed severe muscular hypotonia during the neonatal period prompting exome analysis; however, his hypotonia had resolved spontaneously at six months of age and the family was lost to follow up. Analogous family O was lost to follow up and the outcome of pregnancy of O:II-1 remains unknown. In family B we identified compound heterozygous missense variants; although the inheritance pattern appears plausible, both variants are fairly common with a minor allele frequency (MAF) of 0.1%. Additionally, the phenotype in this patient appears exceptional with the presence of inflammatory changes in the cerebrospinal fluid (CSF) that are not otherwise observed in other subjects herein. For these reasons, these variants have been classified as variants of unknown significance (VUS). A detailed description of all additional patients can be found in the Supplementary information, Figures S1, S2.

### Structural modeling and in silico analysis of Plexin-A1 protein variants

**Fig. 3:**
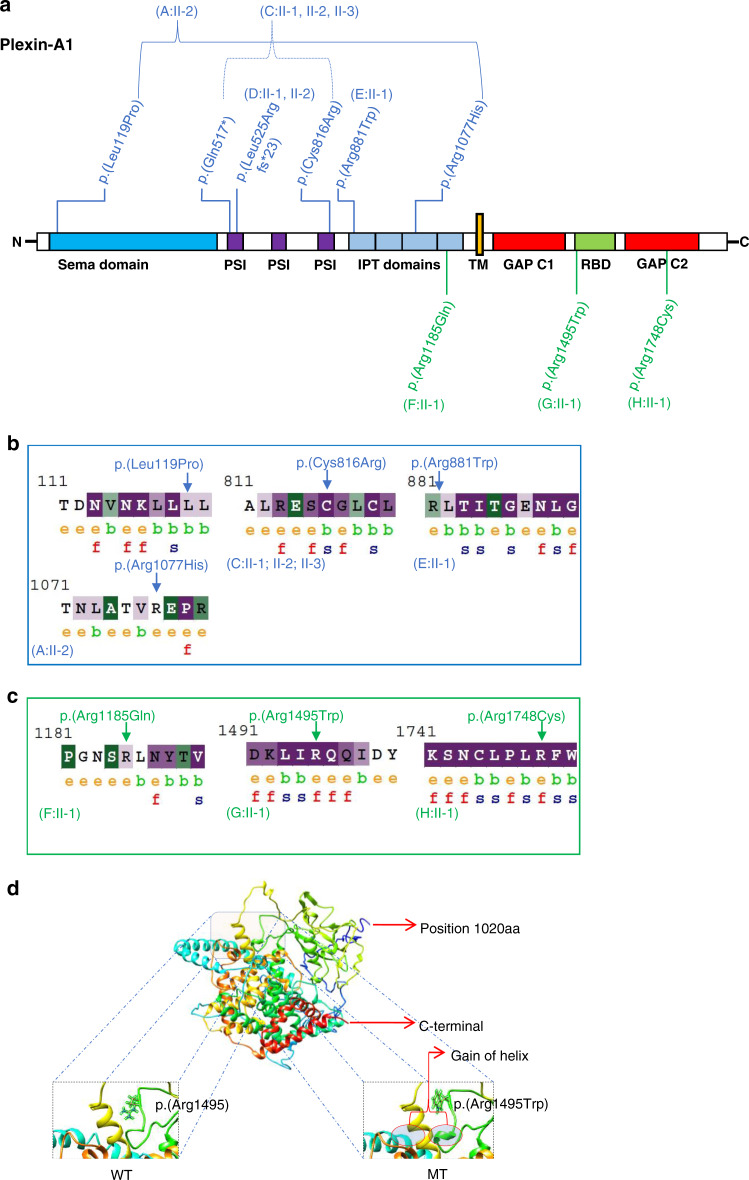
Plexin-A1 variant distribution, conservation, and modeling. **a** Schematic protein domain structure adapted from St. Clair et al.^5^ and localization of Plexin-A1 variants. Note the nonrandom concentration of the biallelic variants (blue) in the extracellular domains and the monoallelic variants (green) toward the intracellular domains. GAP GTPase-activating protein, IPT Ig domain shared by plexins and transcription factors, PSI plexin-semaphorin-integrin domain, RBD Rho GTPase-binding domain, TM transmembrane region. **b**, **c** ConSurf analysis of the biallelic (**b**) and monoallelic (**c**) missense variants. The monoallelic variants (p.[Arg1185], p.[Arg1495], and p.[Arg1748]) are exposed suggesting functional residues in the protein. The intracellular residues p.(Arg1495) and p.(Arg1748) are evolutionarily conserved based on their ConSurf analysis suggesting that these residues react highly sensitively if altered. The biallelic missense variants (p.[Leu119Pro], p.[Cys816Arg], p.[Arg881Trp], p.[Arg1077His]) are noticed to be less conserved compared to the monoallelic variants with the p.(Cys816Arg) variant representing an exemption of this observation. e exposed, b buried, f functional, s structural. **d** 3D protein structure prediction shows replacement of the arginine long side chain by a flat aromatic ring of tryptophan (p.[Arg1495Trp]). Superimposition of the p.(Arg1495Trp) variant onto the wild-type structure showed that there is a gain of helix in the altered protein in close proximity to the site of variant. MT mutated, WT wild type.

The observed distribution of both biallelic variants and monoallelic variants over the Plexin-A1 domains appears to be nonrandom (Fig. 3). Structural models showed a sequence identity of 83%, coverage of 64% and normalized *Z*-score of 3.09. *Z*-score values >1 are considered indicative of correctly folded and good modeled structures and a close approximation of the native structure.^32^ From the structural modeling of mutated Plexin-A1, we observed that all modeled (*n* = 4) biallelic and all modeled (*n* = 3) monoallelic variants likely cause a distortion in the native protein (Figure S3). Superimposition of mutant p.(Arg1495Trp) onto the wild-type structure showed a gain of helix in the mutant protein in close proximity to the variant location (Fig. 3d). The UniProt protein database reports ten putative disulfide bonds in the Plexin-A1 protein. A truncated protein resulting from the p.(Leu525Argfs*23) or the p.(Gln517*) may therefore lack five of those disulfide bonds (515–532; 521–563; 524–541; 535–547; 598–617). In silico analyses using SIFT, CADD, and PolyPhen-2 predicted all monoallelic de novo variants and most biallelic variants to be damaging. ConSurf analysis predicted residues at all positions of the three monoallelic missense variants (p.[Arg1185], p.[Arg1495], and p.[Arg1748]) to be exposed, suggesting that they have a functional role (Fig. 3c). The intracellular residues p.(Arg1495) and p.(Arg1748) are evolutionarily conserved based on their ConSurf analysis suggesting that these residues react highly sensitively if altered.

### Knockdown of *PLXNA1* homologs *plxna1a* and *plxna1b* leads to anomalies of zebrafish central nervous system development

**Fig. 4:**
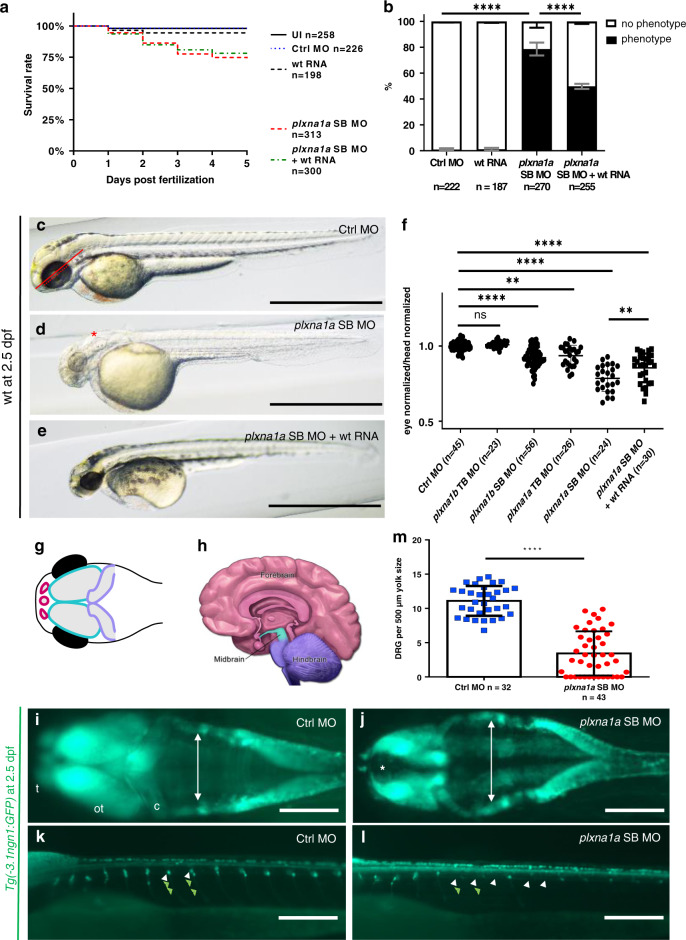
Knockdown of *plxna1a* leads to cerebral anomalies and eye anomalies in zebrafish larvae. **a** Quantification of survival (*N* = 3), zfl injected with *plxna1a* splice blocking Morpholino (SB MO) show a slight but significant reduction (71% with a *p* value <0.0001, two-way analysis of variance [ANOVA]) of survival rate at 5 dpf compared to Ctrl MO (95%) and uninjected zfl (UI, 98%). Survival of *plxna1a* SB MO is not significantly rescued by co-injection of wt *plxna1* RNA (*p* value <0.0001, Mantel–Cox test). **b** The graph shows 100% of surviving zfl at 2 dpf. 79% of *plxna1*a SB MO-injected zfl show a central nervous system (CNS) phenotype as hydrocephalus, smaller head and eye size (*p* value <0.0001, two-way ANOVA, *N* = 3) compared to 0% of UI and 1% of Ctrl MO-injected. The phenotype of *plxna1a* SB MO-injected zfl is significantly rescued by co-injection of wild-type (wt) *PLXNA1* RNA (50% vs. 79%) (*p* value <0.0001, two-way ANOVA). Data are presented as means with standard error of the mean (SEM). **c**–**e** Brightfield images of zfl injected with Ctrl MO, *plxna1a* SB MO, or *plxna1a* SB MO + wt human RNA. Hydrocephalus (asterisk), hypopigmentation, smaller head and eye size are visible. The phenotype of *plxna1a* SB MO-injected zfl (**d**) is partially rescued by co-injection of wt *PLXNA1* RNA (**e**). **f** Eye–head ratio of injected zebrafish larvae at 2 dpf. Measurement of the eye (dotted line) and head (distance between anterior tip up to the otic vesicle) (continuous line) was performed as visualized (**c**). Injection of *plxna1b* SB MO, *plxna1a* TB MO and *plxna1a* SB MO significantly reduced eye–head ratio (**p value 0.0024 or ****p value <0.0001; ordinary one-way ANOVA, *N* = 3), while wt RNA injection in *plxna1a* SB MO-injected zfl significantly rescues the phenotypic effect (*p* value 0.016). Data are presented as means with standard error of the mean (SEM). **g** Schematic of the CNS visible in dorsally mounted *Tg*(*-3,1ngn1:GFP*) zfl at 2 dpf. Pink: forebrain, turquoise: midbrain, purple: cerebellum (part of hindbrain), black: eyes. **h** Schematic of adult human brain (adapted from Midbrain. Blausen Medical. Retrieved on 29 February 2016. http://blausen.com/?Topic=9703). Pink: forebrain, turquoise: midbrain, purple: hindbrain. **i**, **j** *Tg*(*-3.1ngn1:GFP*) zfl are mounted ventral and imaged from dorsal, the anterior to the left. The white arrows mark lateral borders of the hindbrain ventricle and asterisk mark dilatation of the forebrain ventricle (**j**). *plxna1*a SB MO-injected zfl show a dilatation of the ventricle at 2 dpf corresponding to the hydrocephalus seen in brightfield images. (**j**) Note the hypoplasia of telencephalon, mesencephalon, and cerebellum compared to the control (**i**). c cerebellum, ot optic tectum, t telencephalon. **k**, **l** *Tg*(-*3.1ngn1:GFP*) zfl are mounted lateral, anterior to the left. *plxna1*a SB MO-injected zfl have a reduced number of dorsal root ganglions (DRG) (white arrowheads) and corresponding somites lack outgrowing axons (green arrowheads). **m** Quantification of DRG, normalized to yolk size (Figure S6). In *plxna1a* SB MO-injected zfl, the number of DRG is significantly reduced (mean of 3.44 ± 0.49 DRG/500 µm, Ctrl MO 11.1 ± 0.38 DRG/500 µm. *P* value <0.0001, unpaired *t*-test, *N* = 3). White scale bars in all figures: 200 µm. Black scale bars 1,000 µm. **p value <0.01 ****p value <0.0001.

The zebrafish protein Plexin-A1a has a slightly higher amino acid sequence homology with the human Plexin-A1 protein compared to Plexin-A1b (82% vs. 73%, calculated with CLUSTAL_omega from EMBL-EBI). Here, knockdown of *plxna1a* and *plxna1b* in developing zebrafish larvae (zfl) with a splice blocking (SB MO) and a translational blocking morpholino (TB MO) for each of the two paralogs resulted in an overlapping phenotype. Since Plexin-A1a shows the higher homology to human Plexin-A1 and since the knockdown with *plxna1a* SB MO resulted in the most intense phenotype with an only mildly increased mortality (Fig. 4a), we focused on this MO for further analysis. Following the *plxna1a* knockdown, we observed hydrocephalus in midbrain and hindbrain ventricles, generalized hypopigmentation, reduced head size (Fig. 4b, d), reduced eye diameter (Fig. 4d, f), and slightly increased mortality. This phenotype was observed in approximately 80% of *plxna1a* SB MO morphants (*n* = 270) but only in 1% of control MO-injected zfl (*n* = 222, *p* < 0.0001 [two-way ANOVA]) at 2 dpf (Fig. 4b). The measured eye size was normalized to head length to account for variation of embryo size.^31^ This ratio was significantly lower in *plxna1a* SB MO morphants compared to controls (0.31 ± 0.007 vs. 0.48 ± 0.02, *p* < 0.0001 [two-way ANOVA] *N* = 3) (Fig. 4f). Although we observed a reduced head length following the knockdown of *plxna1a* compared to controls, reduction of eye size was still significant after normalization. Efficiency of knockdown with the *plxna1a* SB MO was demonstrated by reverse transcription polymerase chain reaction (RT-PCR) with a decrease of wild-type *plxna1a* expression and presence of an alternative band without exon 5, but no change in *eef1α1* expression as control (Figure S5). Similarly, RT-PCR for the *plxna1b* SB MO confirmed its efficiency (data not shown) as previously demonstrated.^29^

The observed phenotypic spectrum follows the previously published spatiotemporal expression pattern from *plxna1a*/*plxna1b* in situ hybridization studies.^27^ We confirmed the same expression pattern performing immunohistochemistry with an antibody that detects both Plexin-A1a and Plexin-A1b due to their high similarity (Figure S4). Co-injection of human *PLXNA1* RNA together with the *plxna1a* SB MO did not result in a reduction of mortality, but it could significantly rescue the morphologic phenotype (Fig. 4e, b, f).

To assess the impact of the *plxna1a* knockdown on the central nervous system and axonal outgrowth, we used the transgenic *Tg*(*-3.1ngn1:GFP*) reporter line, showing GFP expression in pineal gland, dorsal midbrain, hindbrain, Rohon-Beard sensory neurons, and DRG.^26^ Following the *plxna1a* SB MO injection into *Tg*(-*3.1ngn1:GFP*) zfl, morphants displayed hypoplasia of the telencephalon, mesencephalon, and cerebellum (Fig. 4g, h) as well as dilatation of the ventricles (Fig. 4i, j). Additionally, morphants showed a decrease of migrated DRG cells in the spinal cord above the yolk. The respective somites lack axon outgrowth compared to controls (Fig. 4k–m). To account for variation in embryo size we normalized the DRG count to the yolk sac diameter (Figure S6). Following the *plxna1a* knockdown the number of DRG at 2 dpf was significantly reduced to 3.4 DRG/500 µm (*n* = 32) compared to 11.1 DRG/500 µm in controls (*n* = 43) (*p* < 0.0001 [unpaired *t*-test]) (Fig. 4m, Figure S6).

## DISCUSSION

Neurodevelopmental disorders (NDDs) display extensive genetic and phenotypic heterogeneity.^33^ With the implementation of exome sequencing and family-based rare variant analyses, examples of gene-first/genotype-driven approaches to characterize associated phenotypic spectrums have been illustrated for NDDs.^34,35^ Here, we describe ten patients with NDD ranging from 1.9 years to 42 years from four families with biallelic variants and three families with monoallelic de novo variants in *PLXNA1*. Biallelic and monoallelic variants lead to a phenotypic spectrum primarily affecting the central nervous system. Shared phenotypic features comprise global developmental delay (9/10), congenital anomalies of the brain (6/10), and eye anomalies (7/10). All three patients with monoallelic de novo variants displayed seizures (3/3), but only one of seven patients with biallelic variants (1/7).

The phenotypes observed in *plxna1a* and *plxna1b* zebrafish morphants resemble the phenotypic spectrum we observed in the herein reported patients. *plxna1a* SB MO morphants show a reduced eye diameter when compared to controls (Fig. 4c–f), indicating that *plxna1a* and *plxna1b* are important for eye development analogous to the observed eye phenotypes in our patients. Eye development in zebrafish appears to involve other Plexins as well, since *plxna2* knockdown also leads to reduced relative eye diameter.^5^ Notably, we observed reduced pigmentation in *plxna1a* SB MO morphants compared to controls, which may correlate with the skin abnormalities observed in patients D:II-1, D:II-2, and E:II-1 respectively, presenting with hypo- and hyperpigmented skin anomalies. Another phenotypic feature of the *plxna1a* SB morphants is hydrocephalus (Fig. 4d, j). Here, we report three patients (D:II-1, D:II-2, G:II-1) with an abnormal dilatation of the ventricular system. Additionally, the patient reported by Park et al. showed prominence of ventricles.^16^ *plxna1a* SB MO morphants showed hypoplasia of forebrain, midbrain, and hindbrain obtained in the transgenic reporter fish *Tg*(*-3.1ngn1:GFP*) (Fig. 4j). Accordingly, six of our ten patients presented with structural cerebral anomalies (C:II-2, D:II-1, D:II-2, E:II-1, G:II-1, H:II-1) (Table 1, Fig. 4g, h). The temporal and spatial expression pattern of Plexins in zfl has been studied in detail by Emerson et al., suggesting a dynamic role in neuronal development.^27^ *plxna1a* and *plxna1b* are expressed in the optic vesicle, neural retina, optic tectum, optic chiasm, hypothalamus, medulla oblongata, forebrain, and ventricle of zfl. Here we confirmed this expression pattern (Figure S4). The phenotypic spectrum of *plxna1a* SB MO morphants follows the expression pattern in early development of zfl, suggesting a specific observation. Finally, we detected a decrease of migrated DRG cells in the spinal cord. Consecutively, the axon outgrowth is missing in the respective somites supporting the role of *PLXNA1* as a mediator of axon guidance.^10^ Accordingly, a recent report demonstrated impaired midline crossing of axons in the CC in *Plxna1* knockout mice at E17.5 and agenesis of the CC in newborn mice (P0.5).^11^ Here, we report three patients (C:II-2, D:II-2, G:II-1) with CC anomalies. Analogously, Belyk et al. suggested that polymorphisms in *PLXNA1* are associated with altered developmental trajectory of the CC microstructure.^36^ Additionally, three patients had either signs of congenital cranial neuropathies including sensorineural hearing loss with or without agenesis of vestibulocochlear nerves (C:II-2, G:II-1), unilateral facial palsy (G:II-1), and peripheral axonal neuropathy (D:II-2). These features may be indicative of axonal dysfunction and reflective of the role of *PLXNA1* in axonal guidance. Since the publication of the only two existing *Plxna1* knockout mouse models in 2006 numerous articles describe histopathological abnormalities affecting axonal and neuronal phenotypes. While there are numerous links between the patients reported here and our zebrafish model, others remain without correspondence: for example, it remains unclear whether the *Plxna1*-null mice are developmentally delayed or develop seizures.

Hence, the biallelic and monoallelic variants in *PLXNA1* reported here lead to a distinct overlapping phenotypic spectrum. So far over 30 loci have been linked to disease genes presenting with both recessive (biallelic) and dominant (monoallelic) inheritance patterns.^37^ Investigations of allelic series suggested that allelic heterogeneity may be explained in part by the functional consequences of pathogenic variants, i.e., loss-of-function (LoF), gain-of-function, or dominant-negative mechanisms.^35^ Recently, Harel et al. reviewed 13 of these genes with allelic heterogeneity.^38^ For some of these genes, the gnomAD constraint metric (probability of loss of function intolerance [pLI] score) for loss of function is 0, basically indicating complete tolerance for heterozygous LoF alleles. For example, individuals harboring a heterozygous deletion in *ATAD3A* are unaffected suggesting a dominant-negative pathogenic mechanism or a gain-of-function mechanism for de novo missense variants rather than haploinsufficiency.^39^ However, other genes with reported allelic heterogeneity, e.g., *KIF1A*, *COL6A1*, *ROR2*, and here *PLXNA1* have a pLI score of 1, despite the fact that some healthy parents are heterozygous with LoF variants and affected patients carry monoallelic de novo missense variants. Hence, functional consequences of potentially pathogenic variant alleles alone cannot explain allelic heterogeneity. Interestingly, for *PLXNA1,* gnomAD reports in total 20 individuals with homozygous extracellular and only two individuals with homozygous intracellular missense variants (https://gnomad.broadinstitute.org/). Since the extracellular Plexin-A1 domains comprise 1,245 amino acids and the intracellular domains comprise only 629 amino acids (33%), random distribution of homozygous missense variants should have led to the observation of more intracellular homozygous missense variants. This imbalance might suggest that intracellular missense variants are less tolerated compared to extracellular missense variants. Accordingly, we observed only one patient with a homozygous intracellular missense variant of uncertain clinical significance (K:II-3).

We hypothesize that the here reported biallelic LoF might lead to nonsense-mediated decay (NMD) and the extracellular missense variants lead to impaired dimerization of the Plexin-A1 receptor. Both mechanisms would require two affected alleles in order to affect downstream signaling, whereas the monoallelic intracellular (de novo) missense variants might impair signaling through a dominant-negative effect. Extracellular receptor dimerization and ligand binding may be correct in the intracellular monoallelic situation; however, these variants may harm the dimerized Plexin-A1 receptor macromolecule through a dominant-negative effect in the intracellular domains (Figures S7, S8). However, this concept does not implicate the action of the Plexin-A1 co-receptor NRP1 and other protein–protein interactions of the receptor. Furthermore, two of the previously published monoallelic de novo missense variants reside in extracellular domains of Plexin-A1^16,17^ analogous to the de novo missense variant reported here in patient F:II-1. Finally, we report one de novo LoF variant in a neonate with severe neonatal hypotonia (L:II-3), which resides also in the extracellular domains of Plexin-A1. While the hypotonia resolved spontaneously in this patient, indicating that the identified Plexin-A1 variant might be clinically insignificant, the family was lost to follow up and the outcome remains unknown.

As outlined earlier, binding of semaphorins activates the cytoplasmic GAP domain of Plexin-A1 and alterations of conserved arginine residues in the GAP domain have been shown to diminish this activity.^9^ Previously, Rohm et al. altered three arginine residues—p.(Arg1429), p.(Arg1430), and p.(Arg1746)—of the murine protein in cultured cells.^9^ The murine p.(Arg1746) is the orthologous amino acid residue of the human p.(Arg1748). The data of Rohm et al. suggest that the novel de novo p.(Arg1748Cys) change of patient H:II-1 is functionally abolishing downstream signaling of Plexin-A1, supporting our hypothesis on the pathogenic mechanism of intracellular de novo missense variants in *PLXNA1* (Figure S7). Interestingly, we identified an additional missense variant altering an arginine residue in close proximity to the Plexin-A1 GAP domains (Fig. 3) in patient G:II-1. Hence, the same mechanism described by Rohm et al. for the p.(Arg1748Cys) change may also apply to the additional missense variant identified here. Remarkably, the sequence of the Plexin-A1 GAP domain is highly similar to SYNGAP1, a Ras/Rap GTPase-activating protein that is one of the most frequently mutated genes in pediatric patients with intellectual disability and seizures.^40^ In these children, the disease-causing genetic mechanism is dominant de novo (MIM 612621) with the majority of pathogenic variants in *SYNGAP1* being LoF alleles.^40^ Hence, in support of the above-proposed concept, we might see a dominant-negative effect in patients with monoallelic de novo missense variants leading to impaired downstream GTPase-activating function of the SYNGAP1 related protein Plexin-A1. Notably, all monoallelic variants observed cluster toward the C-terminal domains of Plexin-A1 harboring the two GAP domains (Fig. 3). Analogously, seizures were observed in all three patients with monoallelic de novo variants and only in one patient with biallelic variants.

In conclusion, our study provides evidence that biallelic and monoallelic variants in *PLXNA1* result in a novel neurodevelopmental syndrome mainly comprising developmental delay and brain and eye anomalies.

## Supplementary information

Supplementary Information

## Data Availability

All variants have been deposited into ClinVar (https://www.ncbi.nlm.nih.gov/clinvar/)under Lupski Lab, Baylor-Hopkins CMG, Baylor College of Medicine, including VCV000867235 through VCV000867245.
