# Supplementary material for: Biallelic and monoallelic variants in PLXNA1 are implicated in a novel neurodevelopmental disorder with variable cerebral and eye anomalies
Source: Genet Med. 2021 May 30;23(9):1715–25. doi: 10.1038/s41436-021-01196-9 (PMC8460429; doi:10.1038/s41436-021-01196-9)
Supplement: Supplementary file 1 — Supplementary Information [file 41436_2021_1196_MOESM1_ESM.pdf]

Supplementary information

Supplementary Ethics Statement

Family A: Our study was approved by the Pediatric Ethics Committee of the Tuscany Region (approval no. 2014/0000559), in the context of the DESIRE project (grant no. 602531). We obtained written informed consent for exome sequencing from our patient's family.

Family C: The patients were analyzed in clinical setting, all patients or their families have signed a written consent.

Family D: The study was approved by the Baylor College of Medicine institutional review board (IRB); Research Protocol H-29697.

Family E: This study was approved by the Children’s Hospital at Westmead Human Research Ethics Committee (Biospecimen Bank_10/CHW/45) with informed, written consent from all participants.

Family F: The study has UK Research Ethics Committee approval (10/H0305/83, granted by the Cambridge South Research Ethics Committee and GEN/284/12, granted by the Republic of Ireland Research Ethics Committee).

Family G: The here described study fulfilled the requirement of the Declaration of Helsinki and ethical approval was obtained from the local ethic committee of the Medical Faculty of Bonn (Lfd. Nr. 073/12).

Family H: The patients were analyzed in clinical setting, all patients or their families have signed a written consent.

Family I and Family K: Our patients were consented by UCLH ethics: Project ID: 07/N018; REC Ref: 07/Q0512/26

Family J: This study was approved by the local institutional review board (IRB) (KFSHRC RAC # 2181266).

Family L: This study was approved by the Ethics Committee of the Technical University of Munich (Ethikantrags-Nummer bzw. Votum: #521/16 S).

Family M: Our IRB does not require approval of individual case studies. Our patient signed a written consent allowing release of PHI for publication.

Family N: The study was approved by the Columbia University IRB under protocols AAAA7128 and AAAJ8651.

Family O: The patients were analyzed in clinical setting, all patients or their families have signed a written consent.

Family P: This study was approved by the BC Children's Hospital and University of British Columbia Ethics Board (protocol numberH14-01531). Informed consent was obtained before study inclusion in accordance with the Declaration of Helsinki.

Exome sequencing and data analyses methods

Family A: Trio ES was performed as previously described.[1]

Family C: ES was performed at GeneDx using methods as previously described.[2]

Family D: Exome sequencing (ES) was performed on the individual 1: II-1 and affected sibling (II-2) at the Human Genome Sequencing Center at Baylor College of Medicine (Houston, USA) using methods as previously described.[3] Candidate variants were filtered by comparing minor allele frequencies of variants against publicly available genomic databases, including 1000 Genomes (1000G, http://www.1000genomes.org), Exome Variant Server (EVS, http://evs.gs.washington.edu/EVS), the Atherosclerosis Risk in Communities Study (ARIC, http://drupal.cscc.unc.edu/aric/), Exome Aggregation Consortium (ExAC, http://exac.broadinstitute.org/), genome Aggregation Database (gnomAD, http://gnomad.broadinstitute.org/), and our in-house exome database of ~13,000 individuals at the Baylor College of Medicine Human Genome Sequencing Center. Potential pathogenicity of candidate variants was evaluated using pathogenicity prediction and conservation scores.

Candidate variants with lower conservation scores (PhyloP, GERP++), or those predicted to be tolerated/benign by at least 4 out of 5 pathogenicity prediction tools (FATHMM, MutationTaster, PolyPhen-2, SIFT, PROVEAN) were filtered out.

Family E: Trio ES was performed at the Broad Institute (Boston, USA) as previously described.[4]

Family F: Trio ES was performed as part of the Deciphering Developmental Disorders Study.[5]

Family G: For enrichment of genomic DNA we used the NimbleGen SeqCap EZHumanExome Library v2.0 enrichment kit. For ES a 100bp paired-end read protocol was used according to the manufacturer's recommendations on an Illumina HiSeq2000 sequencer by the Cologne Center for Genomics (CCG), Cologne, Germany. Data analysis and filtering of mapped target sequences was performed with the 'Varbank' exome and genome analysis pipeline v.2.1 as described previously.[6]

Family H: Trio ES was performed on a NextSeq 500 Sequencing System (Illumina, San Diego, CA), with a 2x 150bp high output sequencing kit after a 12-plex enrichment with SeqCap EZ MedExome kit (Roche, Basel, Switzerland), according to manufacturer's specifications. Sequence quality was assessed with FastQC 0.11.5, then the reads were mapped using BWA-MEM (version 0.7.13), sorted and indexed in a bam file (samtools 1.4.1), duplicates were flagged (sambamba 0.6.6), coverage was calculated (picard-tools 2.10.10). Variant calling was done with GATK 3.7 Haplotype Caller. Coverage for these samples was 93% at a 20x depth threshold. Variants were then annotated with SnpEff 4.3, dbNSFP 2.9.3, gnomAD, ClinVar, HGMD, Variome Great Middle East and an internal database. Filtering was performed with criteria based on the consequence on the protein (stop gain, splice site, frameshift indel, in frame indel, missense), frequency in gnomAD (threshold of 3 allele counts for *de novo* variants and 3 homozygous for homozygous and compound heterozygous variants).

Supplementary note: Case reports

Family A: A: II-2 is a 6 year old female of Caucasian descent who exhibited mild developmental delay and had experienced a first febrile seizure at age 15 months and later suffered from nonfebrile seizures and one episode of status epilepticus. Overall, she experienced 15 seizures from age 15 months to 4y7m, all manifested as clonic seizures, and has since been seizure free under phenobarbitone (PB) monotherapy. Although she has been seizure free for almost 18 months, it is uncertain if she benefited from PB medication because some of her last seizures had occurred when she was already being treated with this medication. Her mother (I-2) had a history of a single febrile seizure in early infancy and the proband's older brother (II-1) had also experienced two febrile seizures at age 3 y. Cognitive testing at age 6 years revealed a full scale IQ of 84, with difficulties in executive functions and praxis functions. Psychodiagnostics testing revealed a normal profile. Neurological examination was normal. There was no clinical sign of any hearing impairment.

Family C: C: II-2 is a 4-year-old female of Caucasian descent referred to the Genetics clinic. She showed GDD for her age, is non-verbal and has autistic behaviors. She has severe unilateral sensorineural hearing loss. Physical examination noted ptosis, cupped ears, small teeth, and microcephaly. Brain MRI showed abnormal morphology of the corpus callosum, suggesting dysgenesis or mild presentation of "mega" corpus callosum

Clinical history of the parents noted that the father reported having speech delay that required speech therapy in school and both parents graduated from high school. The proband has two siblings that show similar clinical features. Proband's elder sister (C: II-1) is currently 7 years of age and shows GDD and urinary reflux, younger sister (C: II-3) is currently 21 months of age and also shows GDD and nystagmus.

Family D: D: II-1 is a 12 year old male of Turkish descent that was referred to the Genetics clinic with features of facial dysmorphism and hyperactivity (Figure 1B). He was born at term via normal delivery with a birth weight of 3.2 kg (28th percentile) after an uncomplicated pregnancy. He showed global developmental delays (GDD) from early childhood including motor delay. Clinical

examination at 12 years of age showed normal anthropometric measurements: weight of 51 kg (81st percentile), height of 143 cm (20th percentile) and head circumference of 54.5 cm (69th percentile). Physical examination noted downslanting palpebral fissures, ptosis, sparse lateral eyebrows, flattened nasal bridge, enlarged earlobes, short neck, vertical scar (present since birth and not related to any trauma), and hypopigmented lesions on the torso that measured less than 1 cm (Figure 1C). Diagnostic laboratory work up included metabolic screening, karyotyping, and array CGH which were all reported as normal. Brain MRI revealed dysmorphic ventricular system and prominent Virchow-Robins (perivascular) spaces at the level of the semiovale in both cerebral hemispheres (Figure 1D). Evaluation by a child psychiatrist at 12 years of age showed ADHD, mild autistic features, and an intelligence quotient (IQ) of 77. Ophthalmological evaluation revealed optic disc hypoplasia.

D: II-2 is a 6 year old female (Figure 1E), sister to 1: II-1. She was evaluated for developmental delay/intellectual disability (DD/ID). She was born full term after an uncomplicated pregnancy with a birth weight of 2.8 kg (12th percentile). She did not cry immediately after birth and was in the NICU postnatal for 15 days due to difficulty swallowing and mild respiratory distress. She showed GDD during the first months of life. Growth parameters at 6 years of age showed weight of 16 kg (3rd percentile), height of 113 cm (37th percentile), and head circumference of 51.5 cm (69th percentile). Physical examination showed dysmorphic features including strabismus, sparse lateral eyebrows, flattened nasal bridge, large earlobes, hypopigmented lesions on the torso measuring less than 1 cm (Figure 1F), and foot drop. Laboratory metabolic testing revealed hypothyroidism. Brain MRI showed absent corpus callosum and colpocephalic dilation (Figure 1G,H). Electromyography (EMG) and nerve conduction studies (NCS) were performed due to the observation of foot drop and showed bilateral mild axonal neuropathy involving the peroneal nerves. Array CGH showed a *de novo* 506 kb deletion in the 15q11.2 region, which was absent in the affected brother. Her IQ was evaluated to be borderline normal with a score of 85. Ophthalmological evaluation revealed optic disc hypoplasia, similar to her brother.

Family E: E: II-1 is a 4 year old male of Pakistani descent referred to the clinic with elevated creatine kinase (CK) and autistic features. His early motor and speech development had been normal however his speech had regressed significantly at the age of 2 years and 7 months (went from using 20-30 words consistently to only 1-2 words). He subsequently also developed autistic behavioral features and typical complex motor stereotypies. He was normally grown, not weak and had an age-appropriate gait. He had lower limb hyperreflexia, a hypopigmented macule with an irregular border in the midline of his lower back, a hyperpigmented "streak" along his left arm, and additional hyperpigmented macules on his right temple, and lower back. Brain MRI revealed several high signal foci in the subcortical white matter bilaterally, without associated enhancement or restricted diffusion, with an appearance suggestive of demyelination caused by previous viral infection. He also had normal spinal MRI, cardiac, eye and renal findings/imaging results. He also presented with persistently elevated serum creatine kinase (CK) (2,500 – 3,000 U/L; normal range 200 U/L) with consistently elevated CK also detected in his mother (CK levels 264 U/L) and father (CK levels 782 U/L). Given the history of consanguinity in this family, and high level of homozygosity by decent identified by SNP array (10% AOH spread over 17 autosomal regions: total 288 Mb), it is plausible that individual E: II-1 may have more than one recessive disorder, and that the homozygous *PLXNA1* missense variant p.(Arg188Trp) accounts for his neurodevelopmental phenotype only. DMD MLPA and DM1 test results were normal and prior PathWest gene panel testing (which includes over 300 known neuromuscular genes) had not revealed a diagnosis. Of additional note is the fact that both parents, who are consanguineous, and healthy/not weak also have multiple non-specific pigmentary skin changes. Individual E: II-2 was a spontaneous miscarriage.

Family F: F: II-1 is a female child of unrelated parents. Her mother had previous history of three 1st trimester miscarriages and was diagnosed with epilepsy at the age of 21 years (generalized tonic-clonic seizures) and also had ulcerative colitis. The mother was on Lamotrigine and Mesalazine during the pregnancy. The pregnancy was a natural conception. At the 20 week scan,

the baby was noted to have enlarged cardiac ventricles and there were some concerns about right-sided heart failure with tricuspid regurgitation. For breech presentation she was delivered by elective caesarean section. Preterm delivery at 34 weeks and 6 days of gestation was due to fetal cardiac enlargement. There was also a history of intrauterine growth retardation and she weighed 4lbs at birth. She was intubated and ventilated for two weeks requiring CPAP for a further two weeks. She was a hypotonic baby in the neonatal period. There was significant feeding difficulty requiring NG feeding. There was gastroesophageal reflux and constipation. Episodes of twitching and blank spells started in the neonatal period which were thought to potentially represent seizures. EEG at the time showed cerebral dysmaturity suggestive of cortical dysfunction, frequent sharp wave discharges over the left central region, less frequently in the similar distribution on the right. She was clinically diagnosed with absence seizures. Brain MRI was normal. She was subsequently found to have moderate conductive hearing loss due to a glue ear and was given a softband hearing aid to improve language development. Her generalized hypotonia persisted and there was also joint hypermobility. Cardiac investigations following delivery revealed a dilated cardiomyopathy which was improving on treatment, myxomatous mitral valve with mitral regurgitation were detected. Feeding difficulties persisted. She required PEG feeding but by the age of 7 years was managing to have most of her food orally other than requiring 600ml of high calorie milk to boost her nutritional intake. She sat without support at 9 months. She walked at the age of approximately 2 years 4 months. At 11 months of age her head circumference was 44.5cm (2nd to 9th centile) weight 6.74kg (0.4th to 2nd centile) and height 67cm (0.4th to 2nd centile). At the age of 8-1/2 years, her height was 119.6cm (3rd) and her weight 25.7kg (33rd).

She had evolving picture of significant learning difficulties and now attends special educational needs school at the age of 9 years. She can hold a conversation. She has reasonable comprehension. She knows some numbers and she can copy words. Her particular strength is her memory. She is noted to have a high forehead, mild hypertelorism, extensive joint

hypermobility, hypeperfused palms. Her x-rays showed fusion of her capitate and hamate in the right hand, asymmetrical carpal bone development between the two wrists, slender ribs, narrow iliac wings, hypoplastic pubic rami. She has recurrent dislocation of her elbows.

Family G: G: II-1 was 4.5 years of age at the time of assessment. She has a dizygotic healthy twin sister. They were born prematurely at 33 weeks of gestation. The affected individual presented with GDD, epilepsy, bilateral sensorineural hearing loss aided with bilateral implantation of cochlear implants, impaired vision, unilateral facial palsy, dysmorphic right auricle, dextrocardia, and esophageal atresia with tracheoesophageal fistula, tracheomalacia, and hypocortisolism (Figure 2B). Ophthalmological evaluation revealed optic disc hypoplasia. The EEG was initially normal. Onset of generalized tonic-clonic seizures was observed at three years of age. The Sleep-EEG showed no sharp waves, but generalized intermittent dysrhythmia under treatment with Levetiracetam which was initiated at three years of age. Brain MRI showed enlarged ventricular system, mild thinning of body of CC, delayed myelination, hypoplasia of the brainstem, and agenesis of the posterior pituitary (Figure 2C,D).

Family H: H: II-1 is a 42 year-old female of Caucasian descent. She showed GDD from childhood. She was able to walk at 5 years of age and showed significant speech delay. Currently, she is not able to read or write and her language is limited to a few words or short and simple sentences. She also developed atonic seizures from birth to 12 years of age. Physical examination showed macrocephaly, enophthalmia, and mild face hypotrophy. Other neurological features included spastic paraparesis, and pyramidal signs. Brain MRI showed periventricular leukoencephalopathy, basal ganglia calcifications, and subtentorial atrophy.

Molecular studies

Family A: Trio ES of Patient A: II-2 identified compound heterozygous missense variants in *PLXNA1*. The c.356T>C p.(Leu119Pro) variant was inherited from the mother and the c.3230G>A p.(Arg1077His) from the father. The analysis did not identify additional pathogenic/likely

pathogenic variants in known disease-genes, and *PLXNA1* appeared as a plausible candidate to explain the patient's condition.

Family C: Clinical exome sequencing of individual C: II-2 uncovered compound heterozygous variants in *PLXNA1.* The variants in *PLXNA1* were confirmed to be i*n trans,* with c.1549C>T p.(Gln517*) inherited from the father and c.2446T>C p.(Cys816Arg) from the mother. Additionally, two affected family members, individuals C: II-1 and C: II-3 were compound heterozygous as well for the *PLXNA1* variants. Unaffected sister (twin of C: II-4) isheterozygous for the c.1549C>T variant. The c.1549C>T p.(Gln517*) variant is likely to undergo nonsense-mediated decay.

Family D is part of a cohort of patients with brain malformations and intellectual disability from a predominantly Turkish population. Exome sequencing (ES) analyses of siblings D: II-1 and D: II-2 revealed a homozygous rare variant c.1574_1575del, p.(Leu525Argfs*23) in *PLXNA1* predicted to cause a frameshift deletion in both affected siblings. Sanger sequencing confirmed the variants and segregation according to Mendelian expectations for an autosomal recessive (AR) trait, with both parents showing heterozygosity for the *PLXNA1* variant. Absence of heterozygosity (AOH) analyses from unphased exome data in both siblings demonstrated that the *PLXNA1* variant lies within an ~13Mb AOH region in both siblings. Both siblings, D: II-1 and D: II-2, showed total AOH regions of ~345Mb and ~354Mb respectively, consistent with the reported parental consanguinity. Analysis using NMDEscPredictor tool indicated that the variant is subject to nonsense mediated decay.[7]

Family E: Family E is from a cohort of individuals with features suggestive of a neuromuscular disorder (weakness and/or elevated CK). Exome sequencing of the trio revealed a homozygous variant in *PLXNA1* c.2641C>T p.(Arg881Trp) with both parents showing heterozygosity for the variant.

Family F: Extensive genetic investigations for conditions such as Noonan syndrome, array CGH testing were normal. Individual F: II-1 was recruited to the Deciphering Developmental Disorders Study. Trio ES revealed a heterozygous c.3554C>T, p.(Arg1185Gln) variant in *PLXNA1* in

individual F: II-1. Additionally, a missense variant in the *MAP3K7* gene was identified. Sanger sequencing was performed to confirm that both variants occurred *de novo* in individual F: II-1 and is absent in both parents. Paternity was tested using the Trio ES data.

Family G: Trio ES revealed a heterozygous c.4483C>T, p.(Arg1495Trp) variant in *PLXNA1* in individual G: II-1. Sanger sequencing was performed to confirm that the variant occurred *de novo* in individual G: II-1 and is absent in both parents. Paternity was tested using the Trio ES data.

Family H: Trio ES revealed a heterozygous c.5242C>T, p.(Arg1748Cys) variant in *PLXNA1* in the individual H: II-1. Sanger sequencing was performed to confirm that the variant occurred *de novo* in the individual II-1 and is absent in both parents. Paternity was tested using the Trio ES data.

Rare bi- and monoallelic variants in *PLXNA1* in eight additional families

Family B: B: II-1 is a 12 year old female of Caucasian descent with healthy parents (Figure S1). She showed no developmental delay at the time of assessment, but she developed focal dyscognitive and bilateral tonic-clonic seizures beginning at 7 years of age. Despite treatment with levetiracetam and lamotrigine, she experienced monthly seizures. Cerebrospinal fluid analysis showed inflammatory changes with mild pleiocytosis leading to the suspicion of a CNS-inflammatory disease of unknown origin. However, a brain biopsy of left fronto-parietal region showed focal cortical dysplasia. Brain imaging follow-up showed dynamic focal T2-hyperintense subcortical white matter lesion with contrast enhancement, mild right sided brain atrophy, left fronto-parietal cortical dysplasia with transmantle sign. The study on this family ("Identification and characterization of genetic alterations in rare diseases") was approved by the ethics committee of the University of Leipzig (approval code: 402/16-ek). Exome capture was carried out with Agilent SureSelect All Human Version 6 (60Mb) and the library was then sequenced on a HighSeq sequencer (Illumina) at CeGaT´s laboratory in Tübingen, Germany. Analysis of the raw data was performed using the software Varfeed (Limbus, Rostock, Germany) and the variants were annotated and prioritized using the software Varvis (Limbus, Rostock, Germany). Coverage of 10x was achieved in 98.9% (index), 98.0% (mother), and 99.2% (father) of the targeted sequence. Trio exome sequencing revealed compound heterozygous variants in *PLXNA1*: Chr3, 126733406, NM_032242.3:c.2690G>A, p.(Arg897His) inherited from the father and Chr3, 126708481, NM_032242.3:c.1045G>C, p.(Val349Leu) inherited from the mother. Additional variants in other candidate genes were compound heterozygous variants in *FREM3*: Chr4:144621100-144621101, NM_001168235.1:c.728del, p.(Glu243Glyfs*25) inherited from the father and Chr4:144548788, NM_001168235.1:c.5401C>T, p.(Leu1801Phe) inherited from the mother. Simultaneous follow up *PLXNA1* and *FREM3* through GeneMatcher did not result in

substantial evidence on *FREM3* as a possible disease gene and causality of the variants identified in this family.

Both missense variants were reported in gnomAD with a MAF of 0.1 %, but were not reported in homozygous state. While the patient's phenotype of epilepsy stands within the spectrum of the here reported cohort of patients with *PLXNA1* variants, this case is also exceptional due to the presence inflammatory changes in the cerebrospinal fluid. Furthermore, other clinical features observed within *PLXNA1*-related spectrum (e.g. craniofacial or eye anomalies) are absent in this patient. Taken all of this into consideration, the rather unique phenotype of the patient and common allele frequency of the identified variants in *PLXNA1* led us to classify these variants as VOUS. Identification of additional subjects with these variants either in compound heterozygous state or homozygous state and future functional studies may help clarify the functional impact of these variants.

Family I: I: II-2 is a 2 year-old female child to consanguineous Pakistani parents. The parents and her sister have been reported to be healthy (Pedigree in Figure S1). She showed GDD and non-verbal communication. At eight months of age, she presented with generalized tonic-clonic seizures. Initially she was treated with valproate but seizures continued to occur about four times per month with a duration of approximately five minutes. Levetiracetam was later added and she became seizure-free nine months. The medication was subsequently withdrawn which resulted in early seizures recurrence. She is restless in her behavior. Overall, her trunk musculature is slightly hypotonic (Figure S1B). Brain MRI showed under-opercularization of Sylvian fissures, thinning of the body of CC, concern for cortical dysplasia over precentral gyrus, and white matter signal abnormality consistent with delayed myelination (Figure S1D,E). ES was performed using methods as previously described.[8] ES revealed a homozygous c.231C>G, p.(Asn77Lys) variant in *PLXNA1* in individual I: II-2 (Figure S1C). This missense variant was reported in gnomAD with a MAF of 0.00199 %, but was not reported homozygously. Prediction tools classified the variant to be deleterious (Polyphen-2: probably damaging; SIFT: deleterious; CADD: 23.8). When

performing the segregation we found the patient's father to be heterozygous but the healthy mother and the healthy sister were homozygous for the c.231C>G, p.(Asn77Lys) variant. Thus, c.231C>G, p.(Asn77Lys) cannot explain the phenotype in this family. However, we did not find any other candidate gene from ES and we cannot rule out that it might contribute to a phenotype.

Family J: This consanguineous family has been recently described in a large scale study of more than 2,000 families from Saudi Arabia.[9] In that study, *PLXNA1* has been suggested as a recessive candidate gene for the neurodevelopmental phenotype of the affected child in family J; however, no functional or additional human genetic data supported this finding. Here, we extend the phenotypic description of the affected child of family J. J: II-3 is a boy born full term by normal spontaneous vertex delivery with a birthweight of 3 kg with history of NICU admission for 2 days. The mother noticed that he is delayed in catching his motor milestones at the age of 9 months for which she sought medical advice and was diagnosed as hypotonia. He sat at the age of 1 year and 7 months, crawled at the age of 2 years, started to walk with support at the age of 2 ½ years. He can now speak two-word sentences. He can hold objects and he can transfer from 1 hand to another. He scribbled, used spoon and fork. He has history of 1 admission to hospital for respiratory infection. There is no history of seizure. He is a product of 2nd degree cousin with other 3 healthy children and no family history of similar condition. He is conscious, alert, and interactive. His weight is 8.6 kg below 3rd percentile, height is 86.5 cm on 5th percentile. His head circumference is 48 cm on 25th percentile. There is no apparent cranial nerve palsy. He has decreased muscle bulk and he has generalized hypotonia. Deep tendon reflexes are almost absent. There is no tongue fasciculation. He is weak more distally. Chest, heart and abdominal examinations are unremarkable. Brain MRI showed mild prominence of extra CSF spaces appropriate to his age.

ES revealed a homozygous c.2702G>A, p.(Arg901His) variant in *PLXNA1* in the individual J: II-3. This missense variant was reported in gnomAD with a MAF of 0.0008 %, but was not reported homozygously. Only one of the prediction tools classified the variant to be deleterious (Polyphen-

2: benign; SIFT: benign; CADD: 22.2). However, the parents did not consent for segregation analysis within the family, thereby we cannot ultimately classify this variant as being disease-causing.

Family K: Furthermore, we identified one additional individual with a biallelic variant in *PLXNA1*: The boy was a child to consanguineous Pakistani parents that had two additional unaffected children (Figure S1). Individual K: II-3 showed gross developmental delay and was able to sit at 1.5 years of age. At assessment at 5 years he presented with severe DD, delayed speech, mild nystagmus, brisk reflexes, abnormal movements and he was able to walk with support. He was noted to be aggressive and irritable with features of mild ADHD. Seizures started at one year of age with multiple generalized tonic-clonic seizures per day. Initially seizures in individual K: II-3 were treated with first line drugs including Phenobarbitone and Carbamazepine. Later the regimen was switched to Levetiracetam along with Valproate. His response to treatment was partial. Brain MRI showed abnormal signal intensity in the periventricular white matter bilaterally (hypo-myelination), mild dilatation of the ventricles and particularly the left occipital horn also showed slight wavy margins, thinning of corpus callosum, and generous extra-axial CSF (Figure S1). His response to treatment was partial, seizures persisted and the child recently passed away due to severe pneumonia and status epilepticus.

After informed consent was obtained prior to genetic testing from the family, genomic DNA was extracted from peripheral blood samples according to standard procedures of phenol chloroform extraction. ES was performed as described elsewhere.[10] ES revealed a homozygous c. 4675C>T, p.(Arg1559Cys) variant in *PLXNA1* in individual K: II-3. This variant was confirmed by Sanger sequencing, and both parents as well as the unaffected siblings K: II-2 and II-4 were found to be heterozygous. This variant (g. 126747841C>T) resides in exon 25 and is reported in gnomAD with a MAF of 0.00179%, no homozygous alleles were reported for this variant. The CADD Score for this variant is 31, and Polyphen-2 and SIFT predicted it as being deleterious. Notably, ES revealed a homozygous, p.(Trp233*) variant in *FUCA1* (ENSP00000363603.3) in individual K: II-

3. Biallelic variants in *FUCA1* cause a rare lysosomal storage disease called Fucosidosis [MIM: 230000]. Clinical features include progressive mental (95%) and motor (87%) deterioration, coarse facial appearance (79%), growth retardation (78%), recurrent infections (78%), dysostosis multiplex (58%), angiokeratoma corporis diffusum (52%), visceromegaly (44%), and seizures (38%).[11] An abnormal ventricular system was also reported in two additional individuals with biallelic *PLXNA1* variants including dysmorphic ventricles (family D: II-1) and colpocephaly (family D: II-2). But the abnormal ventricular system observed in K: II-3 (Figure S1) could be also caused by Fucosidosis[12] due to his homozygous nonsense variant in *FUCA1* and the contribution of biallelic *PLXNA1* variant could not be clearly defined. Since, we establish two molecular diagnoses in this individual K: II-3 with a highly overlapping phenotype we cannot draw any conclusion about the contribution of the individual genes. A dual diagnosis, especially in a consanguineous family is not a rare incident.[13]

Family L: The boy was a child to Eritrean parents that had the history of two previous miscarriages (Figure S2A). Because of pathological CTG (cardiotocography), he was born by caesarean section at the 36th week of gestation. Birth parameters are as follows: weight 3.06 kg (21st percentile), birth length: 48 cm (21st percentile), head circumference at birth: 35.5 cm (43rd percentile), APGAR: 3/7/9. Directly after birth, the boy presented with muscular hypotonia. At the age of six months, the muscular hypotonia was no longer present. He had mildly flattened facial profile, ears discretely rotated backwards, and sharp-pointed fingers. Brain MRI was not performed. ES was performed and a NM_032242.3 c.1357C>T p.(Arg453*) *PLXNA1* variant was identified and confirmed by Sanger Sequencing (Figure S2E). The parents were tested for the variant using Sanger sequencing and this variant was not found. Paternity test was performed and *de novo* occurrence could be demonstrated. This variant (g.126710389C>T) resides in exon 2 and is reported in gnomAD with a MAF of 0.003187%, but no homozygous alleles were reported for this variant. The CADD Score is 36. For the truncated p.(Arg453*) Plexin-A1, we removed the

C-terminal beyond p.(Arg453) residues and subjected the rest of the sequence to I-Tasser based modeling. The resulting model can be seen in Figure S3.

Since the family has been lost to follow up, we had no information about further development of the child (e.g. history of development delay, seizures, or cerebral anomalies). However, the sponatenously resolved neonatal hypotonia remains unclear. Thus, we cannot classify this variant as being disease-causing.

Family M: The child of African American and African American/Caucasian parents was adopted. At last exam he was 8.5 years of age. He showed GDD and ASD. Brain MRI showed occasional punctate junctional hyperintensities in the anterior cerebrum of doubtful significance. He developed generalized convulsive seizures at age of 7 years and was started on ethosuximide and after increasing the dose he was seizure free. His EEG showed rare generalized epileptiform discharges/activity as well as multifocal fragmentary discharges. He had tracheomalacia requiring tracheostomy and an absent left kidney was noted. There was suspicion of fetal alcohol spectrum disorder. A chromosomal microarray showed arr Xp11.23(47,668,529-48,193,565)x2 mat, 4q21q22.1(88,102,193-88,599,364)x3 *de novo*. These results however have been interpreted as benign findings. Later, ES revealed a heterozygous c.2150 T>A, p.(Val717Glu) variant in *PLXNA1* (Figure S2C). This variant was not reported in gnomAD. Prediction tools classified the variant to be deleterious (Polyphen-2: possibly damaging; SIFT: deleterious; CADD: 25.2). However, parental samples were not available for segregation analysis. An additional molecular finding in this patient was a missense variant in *TBC1D1* c.2673 A>C, p.(Gln891His) VUS, which may contribute to the renal anomalies.[14] Since we were not able to confirm the *de novo* occurrence in this family, we cannot classify the *PLXNA1* variant as being disease-causing.

Family N: We present a 45-year-old male patient with a history of intellectual disability, developmental delay, and congenital ptosis and cataract (Figure S2A). He was born full term following an unremarkable pregnancy. He had delayed developmental milestones, including speech acquisition between three and four years of age, and required special education

throughout his school years. He developed temporal lobe epilepsy in the third decade of life. He then developed asymmetric levodopa-responsive parkinsonism at the age of 38, characterized by right limb rigidity and bradykinesia. His clinical course was complicated by peak dose dyskinesias and bothersome off dystonia. By the age of 45, his parkinsonian symptoms and levodopa-induced dyskinesias progressed significantly. The same year, he died in his sleep and the cause of death was unknown, although cardiac etiologies were considered.

The patient was tested for pathogenic variants in *RAB39B*, and results were negative. He had a maternal family history of orthostatic tremor and a paternal family history of essential tremor, but he did not have a family history of Parkinson's disease or parkinsonism. He was of European ancestry. Brain imaging throughout his adult life revealed findings suggestive of mesial temporal sclerosis (MTS) (Figure S2D,F,G).

ES revealed a heterozygous c.3361G>T, p.(Glu1121*) variant in *PLXNA1*. This variant was not reported in gnomAD. Prediction tools classified the variant to be deleterious (CADD: 41). This stop-gain variant was confirmed in Sanger sequencing and was not found in the patient's father, but the mother was not available for segregation analysis (Figure S2E). Since we were not able to confirm the *de novo* occurrence in this family, we cannot classify this variant as being disease-causing.

Family O: The fetus was 20 weeks of gestation at time of assessment (Figure S2 A). Prenatal ultrasound showed square appearance of the frontal horns and thick corpus callosum. The outcome of this pregnancy remains unclear at the time of writing. ES revealed a heterozygous c.3883G>A, p.(Glu1295Lys) variant in *PLXNA1*. This missense was found to be *de novo*. This missense variant was not reported in gnomAD. Prediction tools classified the variant to be deleterious (Polyphen-2: possibly damaging; SIFT: deleterious; CADD: 31). Because of the very limited data, we cannot draw sufficient genotype-/ phenotype-correlations and report this patient in the supplementary information.

Family P: Individual II-1 was identified through the Epilepsy Genomics Study (EPGEN) at BC Children's Hospital, a clinical study assessing the utility and economy of targeted ES in children with early-onset epilepsy of unknown cause.[15] ES was performed with the Ion AmpliSeq Exome Kit (57.7 Mb) and Ion Proton System (Life Technologies, Carlsbad, CA). Analysis was restricted to 620 genes previously implicated in epilepsy.

P: II-1 is currently a 14 year-old male of Asian descent. He showed gross and fine motor delays from childhood and has an abnormal or clumsy gait. He was diagnosed with autism spectrum disorder and oromotor dyspraxia. Although he was not formally diagnosed with intellectual disability, he does require learning assistance at school. He also showed abnormal sleep patterns, he requires a nap every day for an hour and then sleeps for 11-12 hours during the night. He had seizure onset at 10 months of age, initially his seizure type was hemi-clonic and it occurred occasionally, usually with fever as a trigger. He remained seizure-free from 5-9 years of age, after which, he had focal seizures with impairment of consciousness or awareness. His current anti-epileptic drugs (AEDs) are Perampanel and Eslicarbazepine. He failed on four other AEDs and had partial response to VNS. Interictal EEG demonstrated dysrhythmic background and generalized and multifocal epileptiform activity including generalized sharp and slow wave activity. During a typical head drop seizure, ictal EEG showed a generalized delta wave followed by generalized suppression and bifrontal delta. His physical examination showed mild dysmorphisms in the form of ear tags. He has a myopic refractive error starting at age of 14, corrected with prescription glasses. Brain MRI was noted to be normal at 11 years of age.

ES revealed a heterozygous c.5632C>T, p.(Arg1878Trp) variant in *PLXNA1* (Figure S2H). This missense variant was reported in gnomAD with a MAF of 0.00199 %, but was not reported homozygously. Prediction tools classified the variant to be deleterious (Polyphen-2: possibly damaging; SIFT: deleterious; CADD: 25.2).

This variant was confirmed using Sanger sequencing and was found to be absent in the unaffected mother. However, paternal DNA was unavailable for testing. Another variant of interest

was found in *SETD2* c.5781G>T, p.(Gln1927His) which was similarly absent in the mother. Since we were not able to confirm the *de novo* occurrence in this family, we cannot classify this variant as being disease-causing.

Supplementary Figures

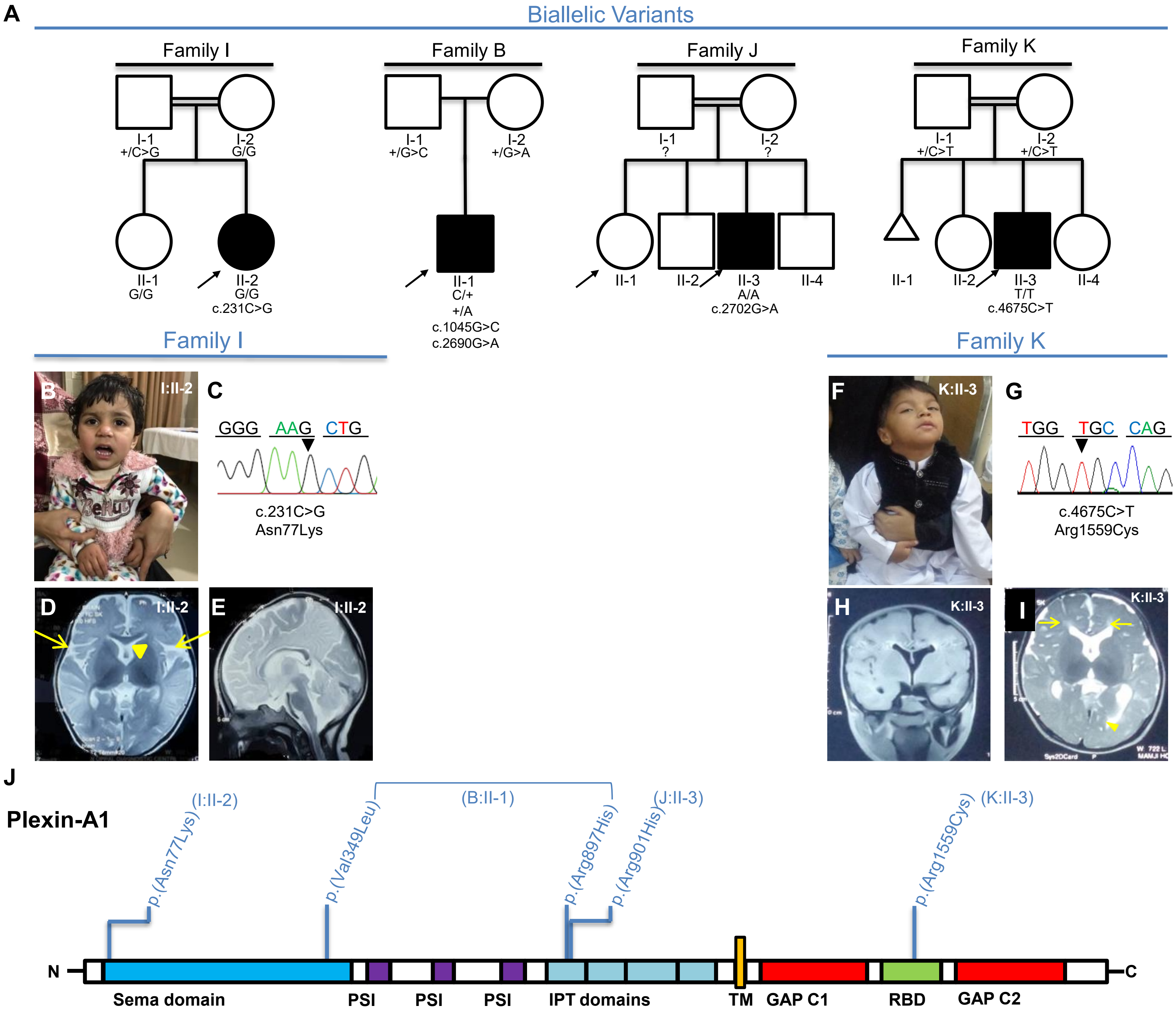

A
Biallelic Variants
Family I
I-1
+/C>G
I-2
G/G
II-1
G/G
II-2
G/G
c.231C>G
Family B
I-1
+/G>C
I-2
+/G>A
II-1
C/+
+/A
c.1045G>C
c.2690G>A
Family J
I-1
?
I-2
?
II-1
II-2
II-3
A/A
c.2702G>A
II-4
Family K
I-1
+/C>T
I-2
+/C>T
II-1
II-2
II-3
T/T
c.4675C>T
II-4
Family I
B
I:II-2
C
GGG AAG CTG
c.231C>G
Asn77Lys
D
I:II-2
E
I:II-2
Family K
F
K:II-3
G
TGG TGC CAG
c.4675C>T
Arg1559Cys
H
K:II-3
I
K:II-3
J
Plexin-A1
(I:II-2)
p.(Asn77Lys)
p.(Val349Leu)
(B:II-1)
p.(Arg897His)
p.(Arg901His)
(J:II-3)
p.(Arg1559Cys)
(K:II-3)
N
C
Sema domain
PSI
PSI
PSI
IPT domains
TM
GAP C1
RBD
GAP C2

Figure S1. Pedigrees, facial images and brain MRI results of families with biallelic variants in *PLXNA1* of unknown significance.

(A): Pedigrees of three consanguineous families with biallelic variants in *PLXNA1*.

(B): Family I: II-2 without any obvious dysmorphic features.

(C): Sanger sequencing results of Family I: II-2. Note that I: I-2 and I: II-1 are also homozygous for the c.231C>G *PLXNA1* variant.

(D): Family I:II-2: Axial T2 MRI showing under-opercularization of the Sylvian fissures (yellow arrows), borderline thinning of the CC, and delayed myelination for age (as evident by white matter hyperintensities, thinning of the anterior limb of the internal capsule [arrow head]).

(E): Family I: II-2: Sagittal T2 MRI showing wide Cingulate sulcus, generous extra-axial spaces, thinning of the body of CC, and concern for cortical dysplasia at the pre-central gyrus.

(F): Family K: II-3 with mild coarse facial features likely related to his diagnosis of Fucosidosis.

(G): Sanger sequencing results of Family K: II-3.

(H),(I): Family K: II-3. Brain MRI showed abnormal signal intensity in the periventricular white matter bilaterally (hypo-myelination, yellow arrows), mild dilatation of the ventricles and particularly the left occipital horn also showed slight wavy margins (arrow head), thinning of corpus callosum, and generous extra-axial CSF.

(J) Schematic protein domain structure adapted from St.Clair *et al.*[16] and localization of biallelic variants in Plexin-A1 of unknown significance. Note that the variants of Family I: II-2 and J: II-3 reside in the extracellular domain, in accordance with the biallelic variants presented in the main text. PSI: plexin-semaphorin-integrin domain; IPT: Ig domain shared by plexins and transcription factors; TM: transmembrane region; GAP: GTPase activating protein; RBD: Rho GTPase-binding domain.

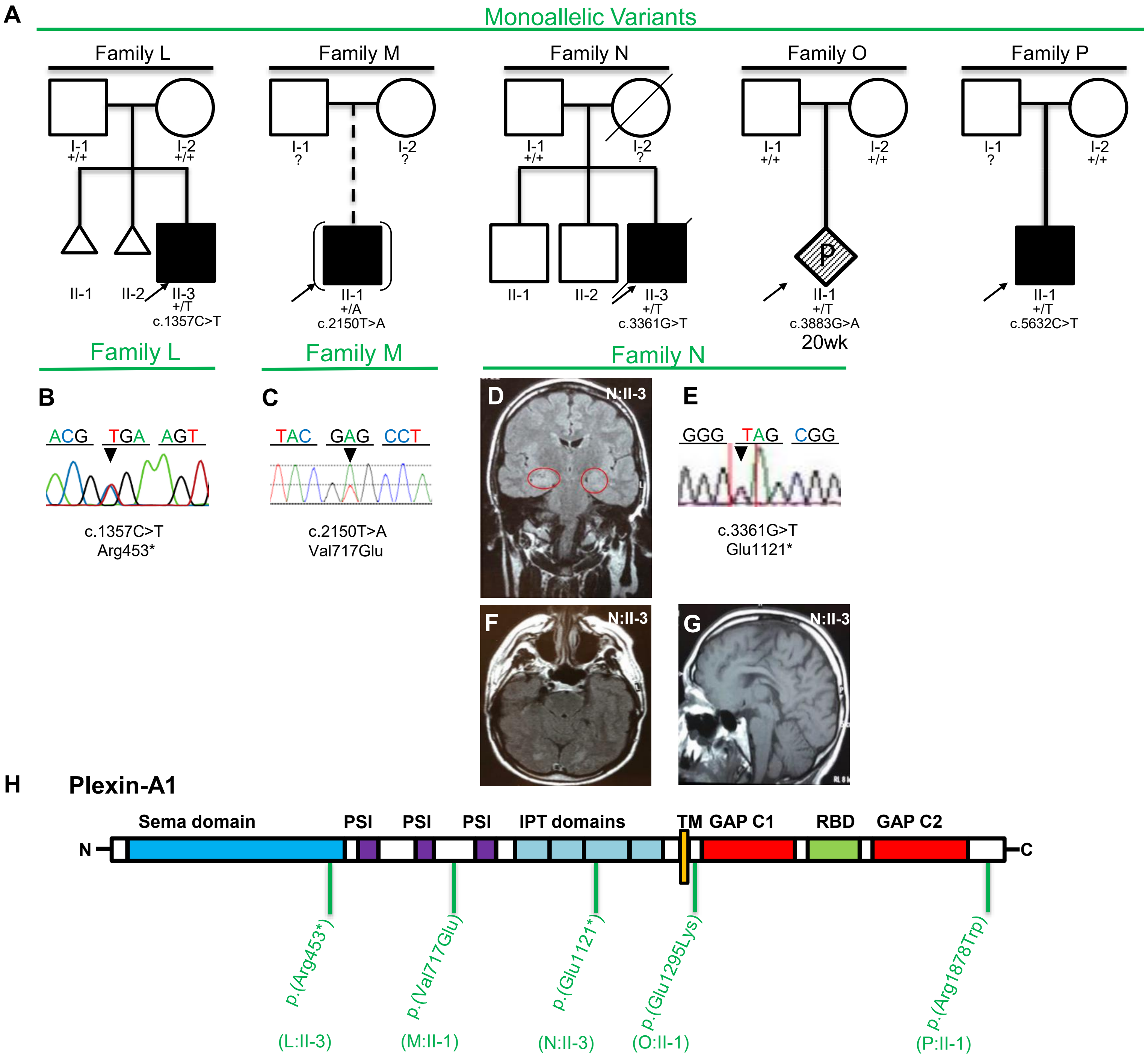
A
Monoallelic Variants
Family L
I-1
+/+
I-2
+/+
II-1
II-2
II-3
+/T
c.1357C>T
Family M
I-1
?
I-2
?
II-1
+/A
c.2150T>A
Family N
I-1
+/+
I-2
?
II-1
II-2
II-3
+/T
c.3361G>T
Family O
I-1
+/+
I-2
+/+
P
II-1
+/T
c.3883G>A
20wk
Family P
I-1
?
I-2
+/+
II-1
+/T
c.5632C>T
Family L
B
ACG TGA AGT
c.1357C>T
Arg453*
Family M
C
TAC GAG CCT
c.2150T>A
Val717Glu
Family N
D
N:II-3
E
GGG TAG CGG
c.3361G>T
Glu1121*
F
N:II-3
G
N:II-3
H
Plexin-A1
Sema domain
PSI
PSI
PSI
IPT domains
TM
GAP C1
RBD
GAP C2
N
C
p.(Arg453*)
(L:II-3)
p.(Val717Glu)
(M:II-1)
p.(Glu1121*)
(N:II-3)
p.(Glu1295Lys)
(O:II-1)
p.(Arg1878Trp)
(P:II-1)

Figure S2. Pedigrees and brain MRI results of families with monoallelic variants in *PLXNA1* of unknown significance.

(A): Pedigrees of five families with monoallelic variants in *PLXNA1*.

(B), (C), (E): Sequencing results of Family L: II-3, Family M: II-1 and Family N: II-3.

(D), (F), (G): Family N: II-3: Brain MRI showing prominence of cerebellar folia, signal abnormality within the parahippocampal formations associated with volume loss (i.e. mesial temporal sclerosis, red circles), possible volume loss in the right parietal region (at age 34 years).

(H): Schematic protein domain structure adapted from St.Clair *et al.*[16] and localization of monoallelic variants in Plexin-A1 of unknown significance. PSI: plexin-semaphorin-integrin domain; IPT: Ig domain shared by plexins and transcription factors; TM: transmembrane region; GAP: GTPase activating protein; RBD: Rho GTPase-binding domain.

Plexin-A1 protein modeling monoallelic variants

Plexin-A1 WT

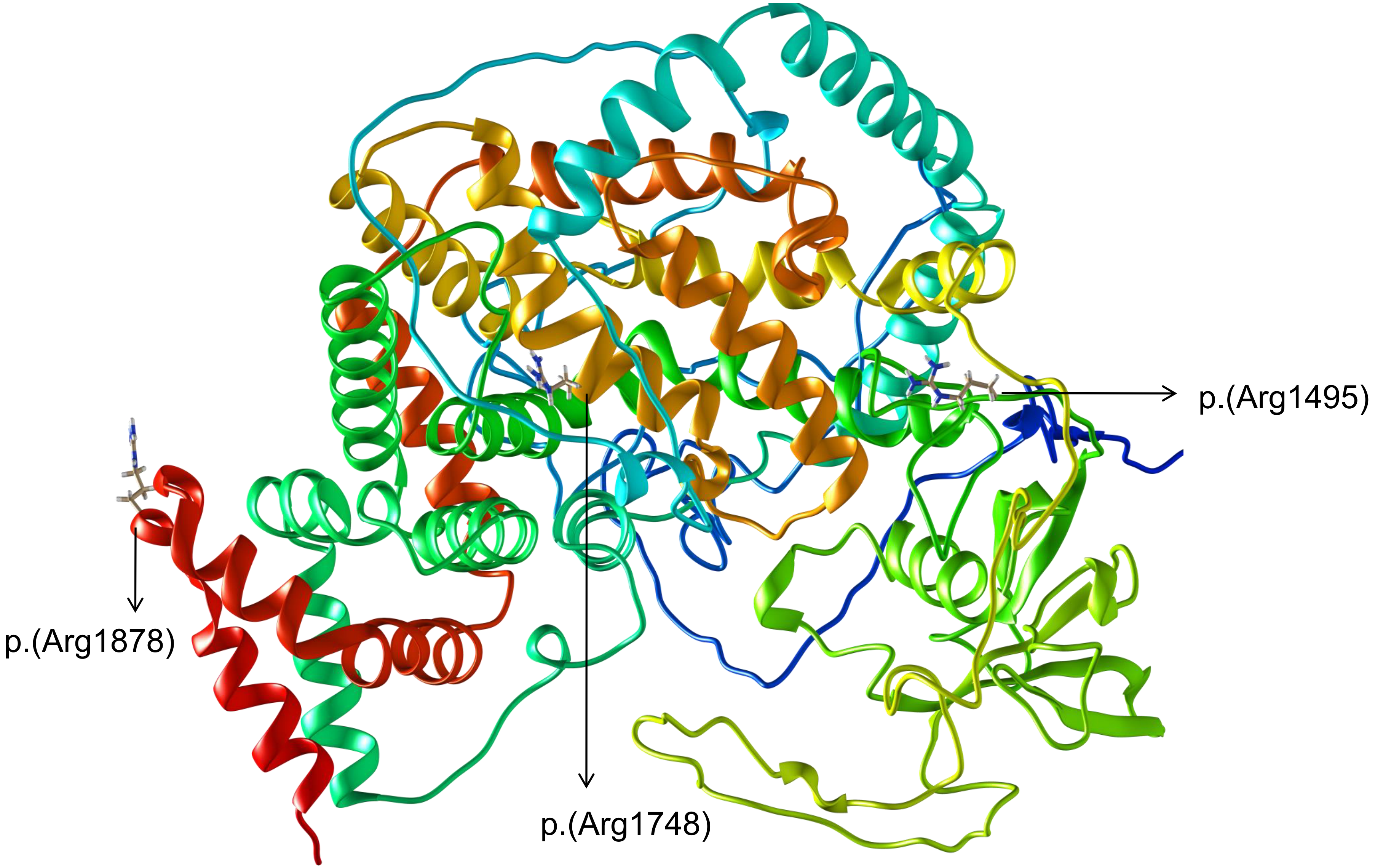

Family F

Plexin-A1 WT versus p.(Arg1185Gln)

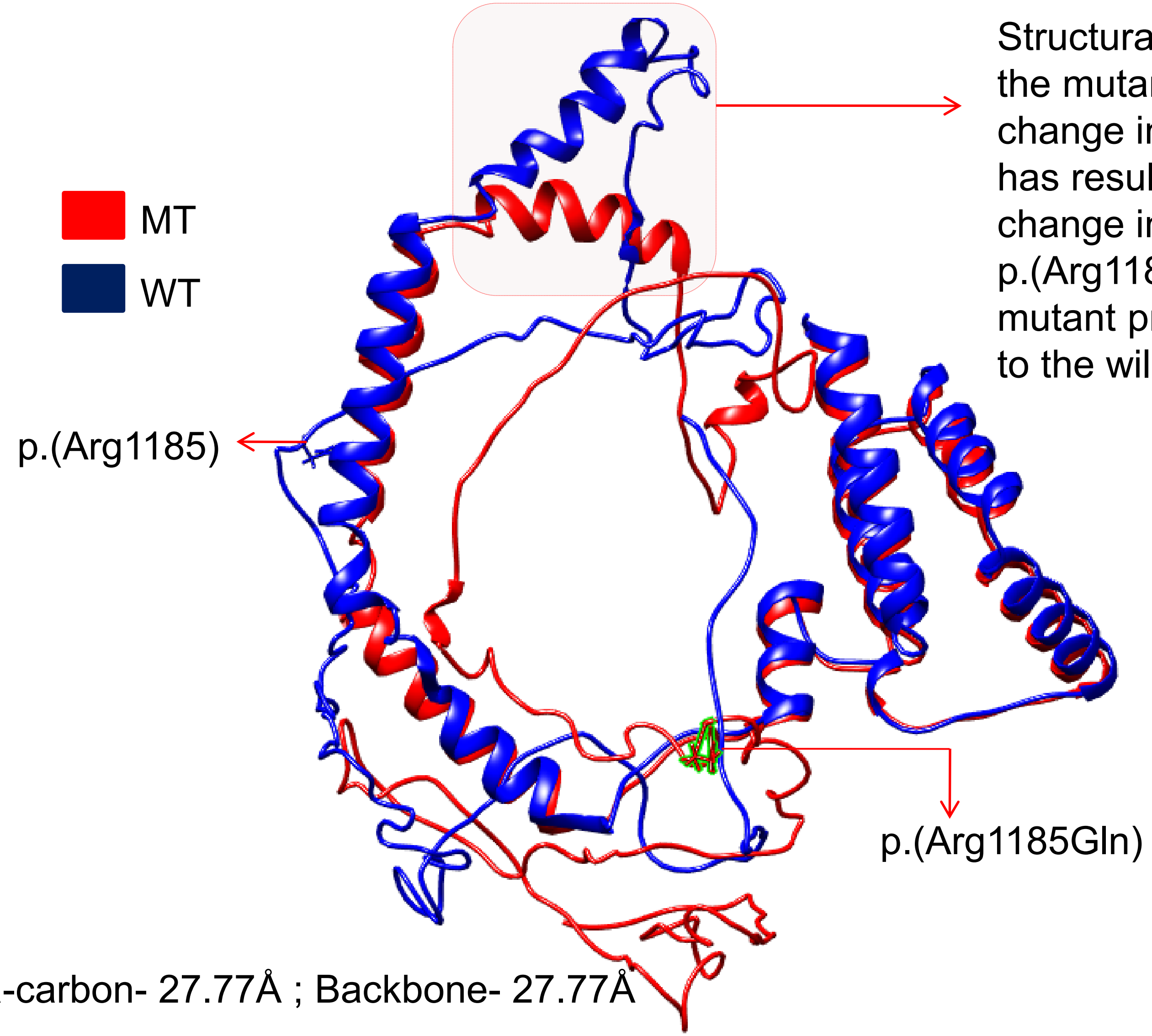

Structural deformation in the mutant protein due change in helix positioning has resulted in drastic change in the position of p.(Arg1185Gln) in the mutant protein compared to the wild type

RMSD: Alpha-carbon- 27.77Å ; Backbone- 27.77Å

Family G

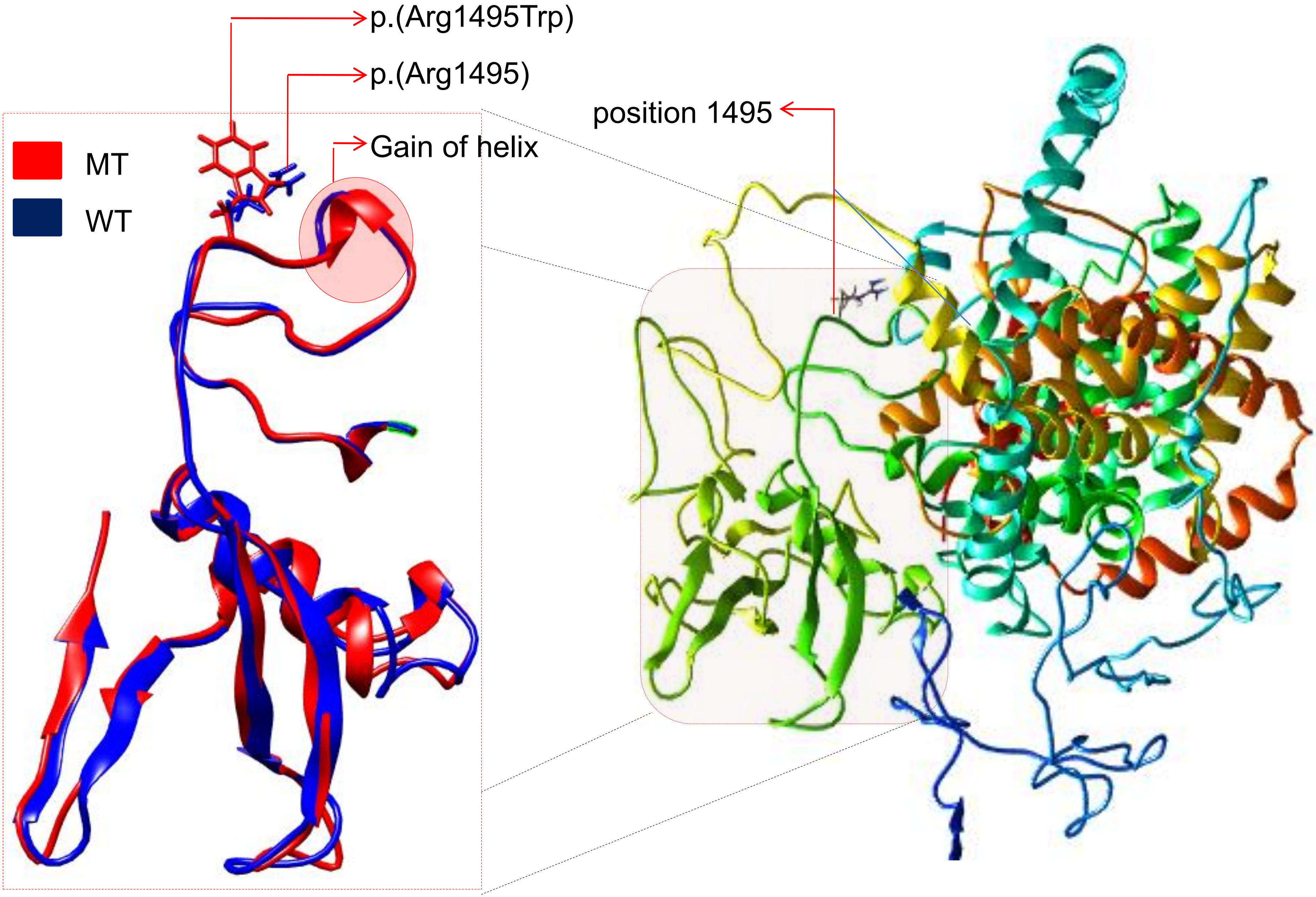

RMSD: Alpha-carbon- 0.28Å ; Backbone- 0.63Å

Family H

Plexin-A1 WT versus p.(Arg1748Cys)

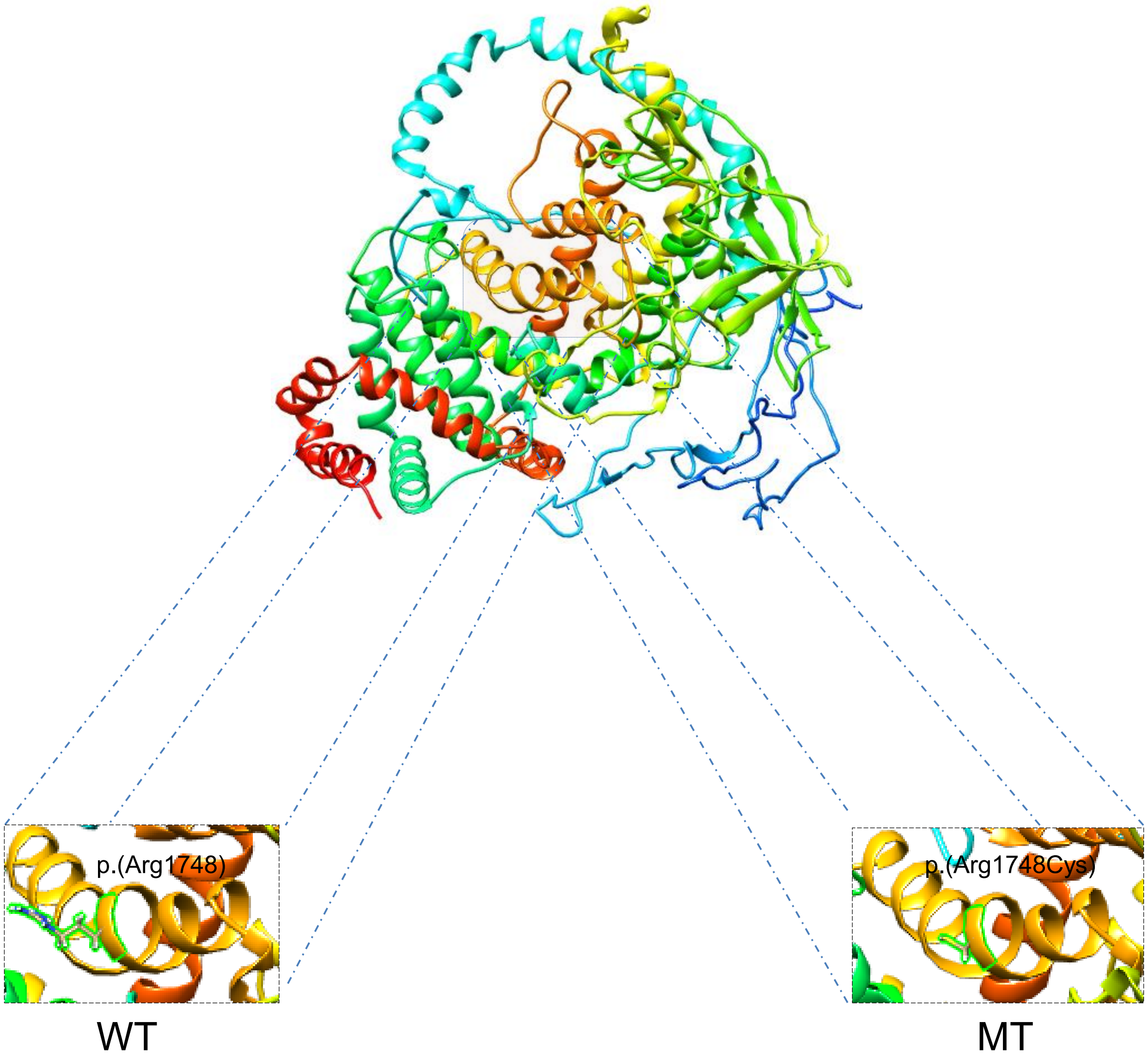

Family H

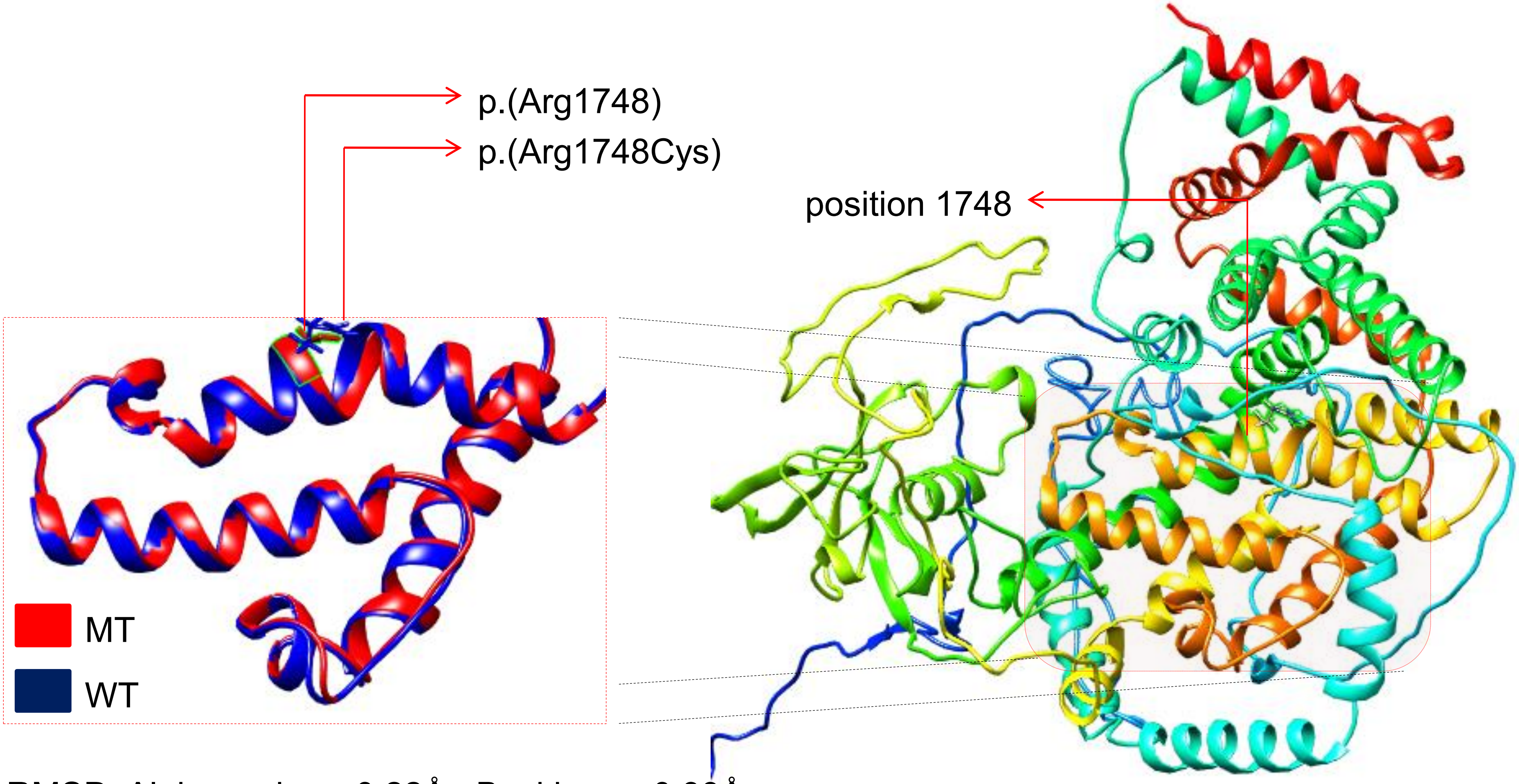

RMSD: Alpha-carbon- 0.22Å ; Backbone- 0.39Å

Plexin-A1 protein modeling biallelic variants

Family D

Plexin-A1 p.(Lys525Argfs*23)

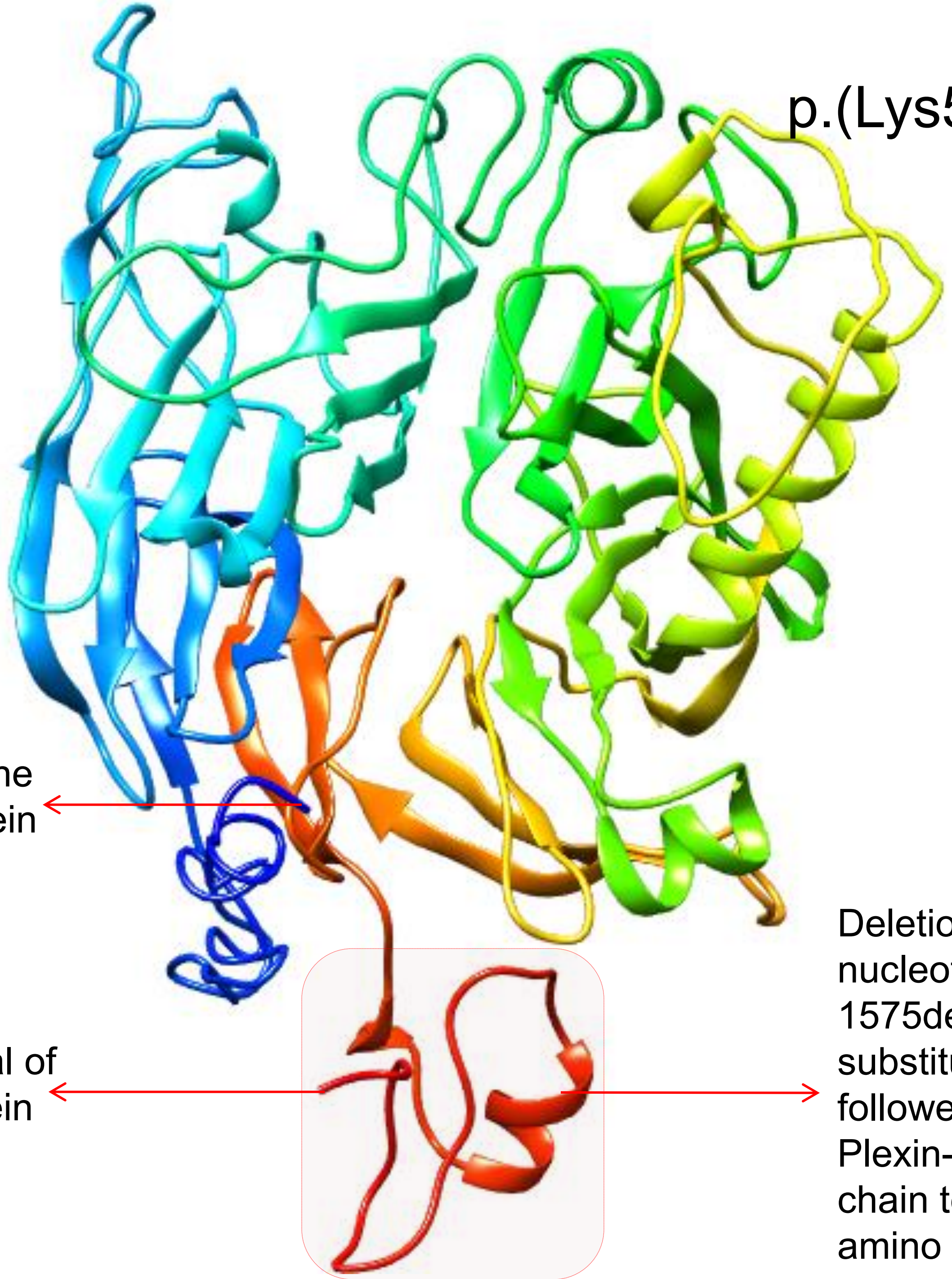

N-terminal of the Plexin-A1 protein

Truncated C-terminal of the Plexin-A1 protein

Deletion of two nucleotides (c.1574_ 1575del) resulted in a substitution p.(Leu525Arg) followed by frameshift in Plexin-A1 protein until chain termination after 23 amino acids.

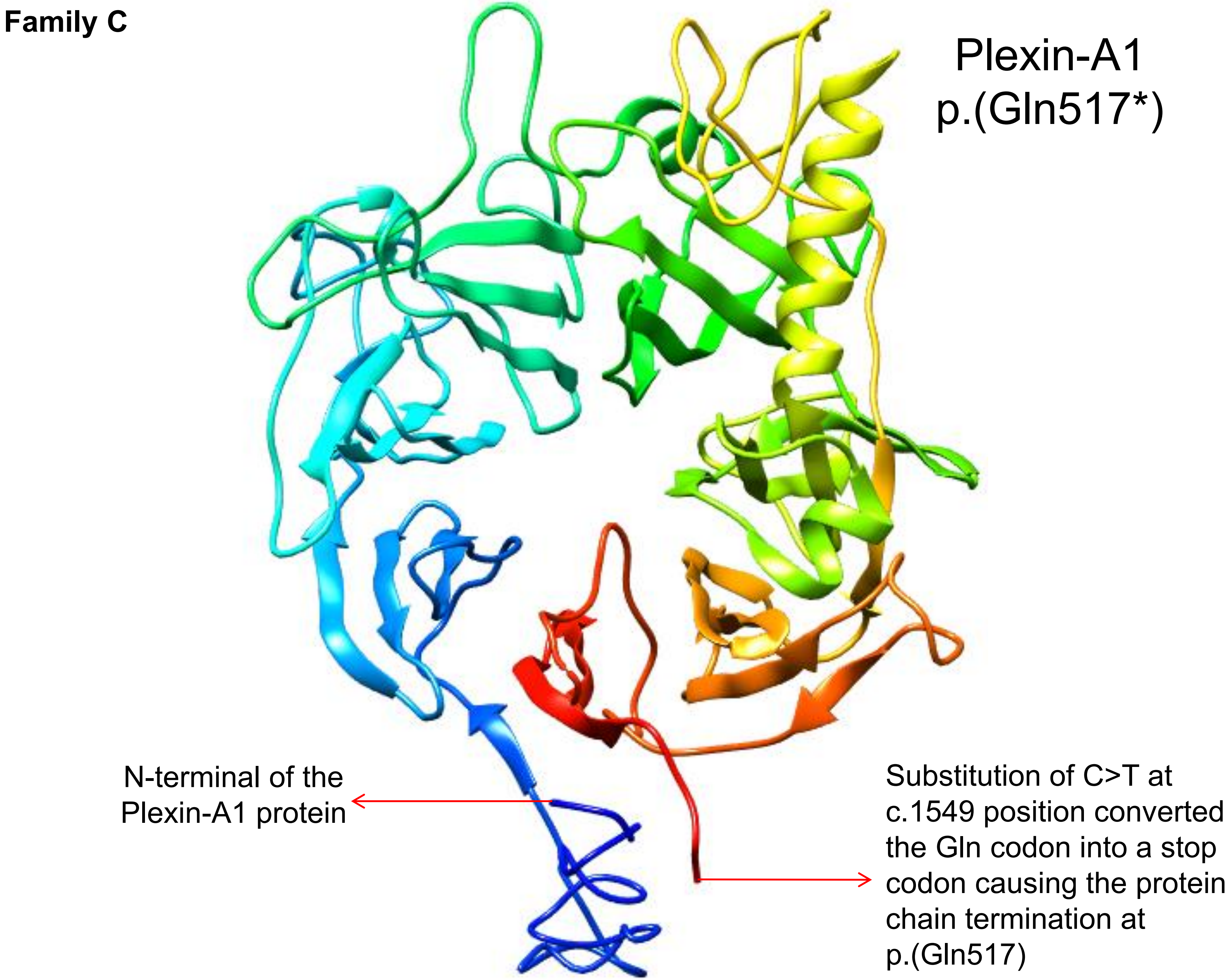
Family C
Plexin-A1
p.(Gln517*)
N-terminal of the Plexin-A1 protein
Substitution of C>T at c.1549 position converted the Gln codon into a stop codon causing the protein chain termination at p.(Gln517)

Family C

Plexin-A1 p.(Cys816Arg)

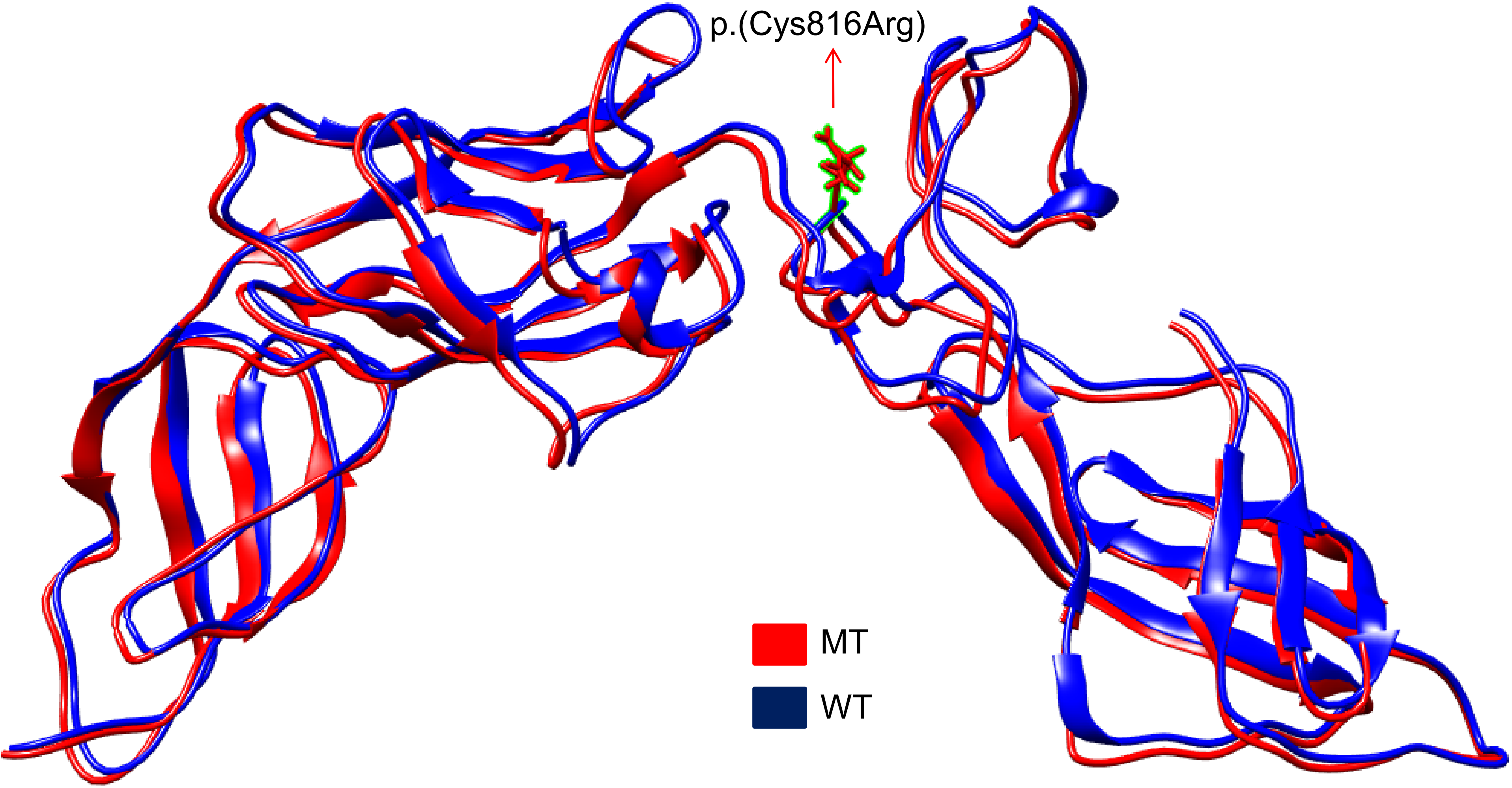

RSMD: Alpha-carbon- 0.44Å ; Backbone- 0.74Å

Family E

Plexin-A1 p.(Arg881Trp)

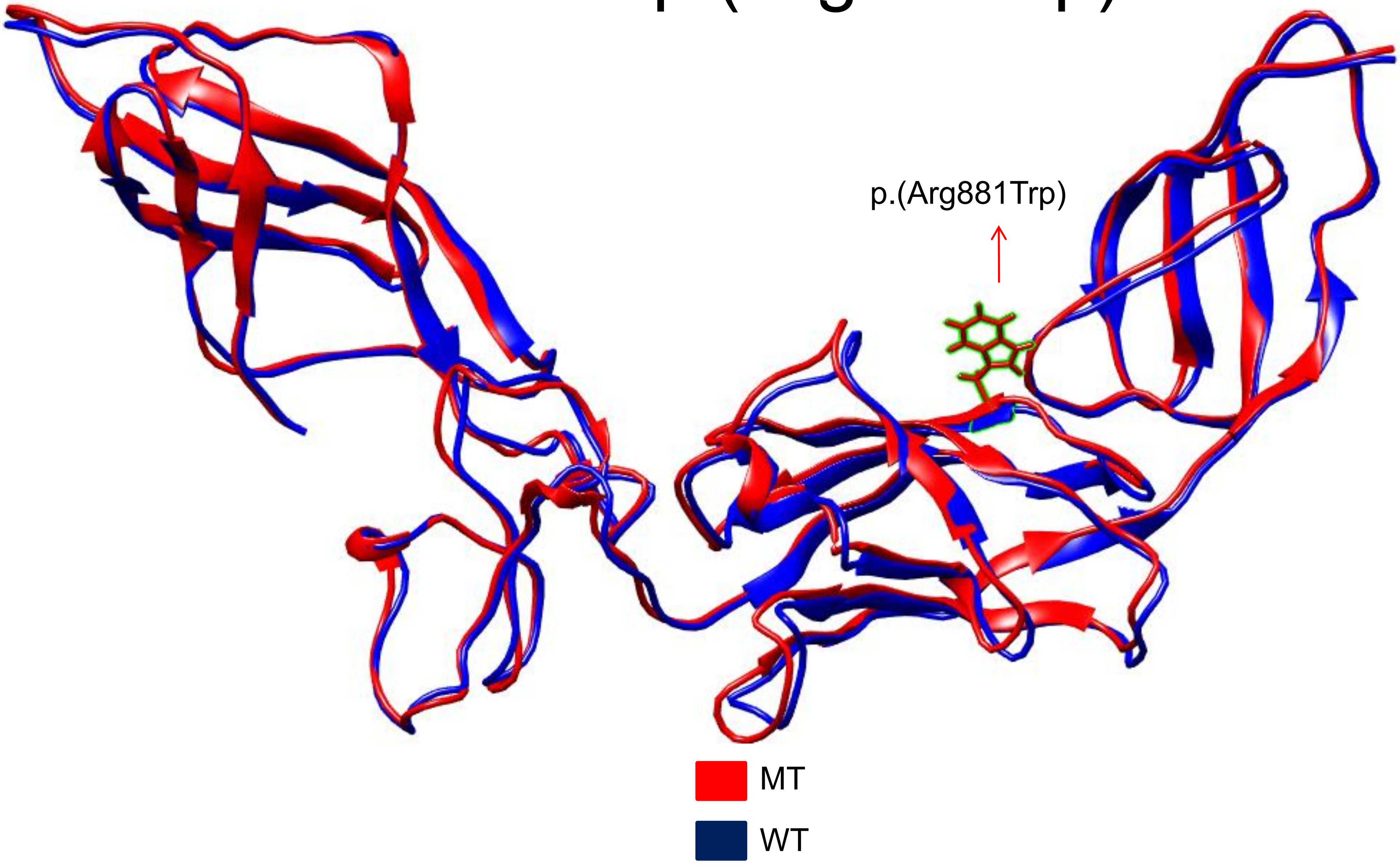

RSMD: Alpha-carbon- 0.44Å ; Backbone- 0.74Å

Figure S3. Protein structure modeling of variants in Plexin-A1.

The 3D protein structural models were built using I-Tasser. A detailed description is given in Materials and Methods.

Plexin-A1a/Plexin-A1b Immunostaining at 3 dpf

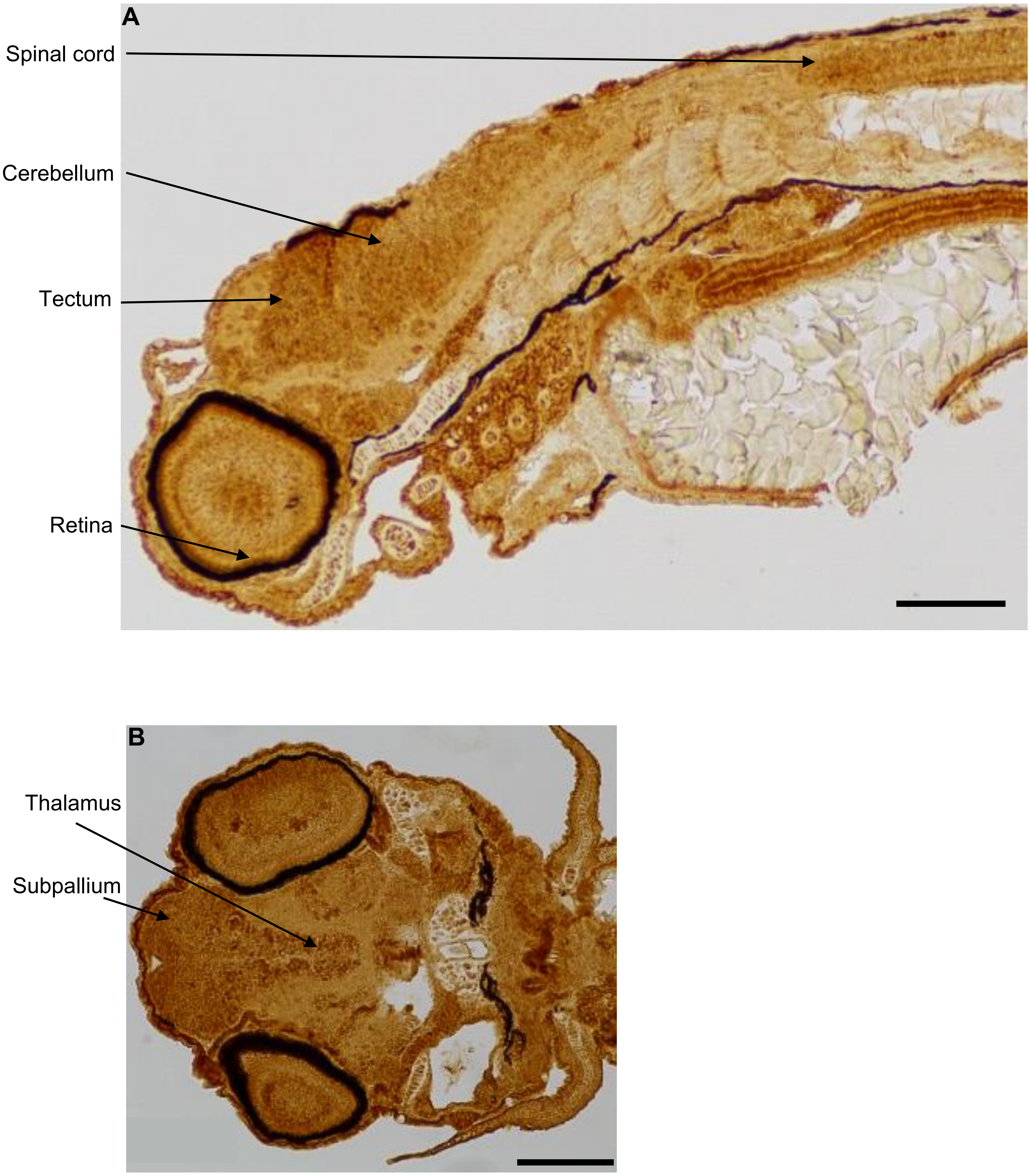

Figure S4. Immunohistochemistry

Immuno-histological staining against Plexin-A1a and Plexin-A1b using an anti Plexin-A1 antibody (AbCam; ab23391) on 5 µm thick paraffin sections of a 3 dpf zfl.

(A) Sagittal section of the cranial half of the zfl showing Plexin-A1a/b positive cells e.g. in the retina, optic tectum, cerebellum and spinal cord.

(B) Frontal section of the head of the zfl additionally showing Plexin-A1a/b positive cells e.g. in the subpallidum and the thalamus region.

Scale bar in (A) 200 µm, in (B) 100 µm.

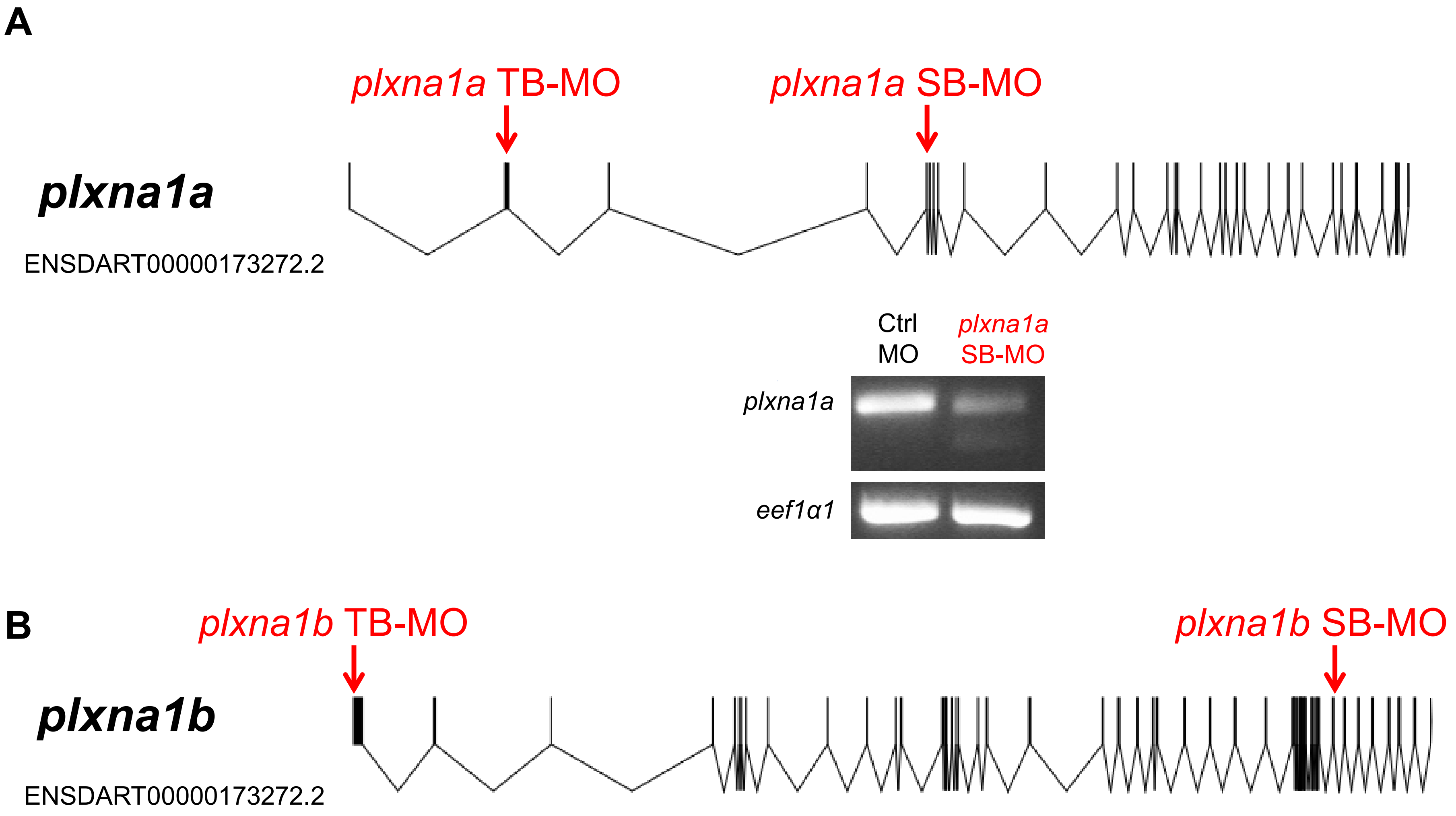
A
plxna1a TB-MO
plxna1a SB-MO
plxna1a
ENSDART00000173272.2
Ctrl MO
plxna1a SB-MO
plxna1a
eef1α1
B
plxna1b TB-MO
plxna1b SB-MO
plxna1b
ENSDART00000173272.2

Figure S5. Exon structure of zebrafish *plxna1a* and *plxna1b* with morpholino targets

(A) Schematic of *plxna1a* gene, depicting the target sites of the translational blocking (TB MO) and the splice blocking Morpholinos (SB MO) (red). The *plxna1a* SB MO is overlapping the exon 5 splice donor site.

RT-PCR was performed with *plxna1a* forward primer overlapping Exon 3 and 4 and reverse overlap Exon 6 and 7 and therefore only cDNA was amplified. Picture of Ethidium-bromide stained gel after performing RT-PCR with zfl harvested at 2 dpf. *plxna1a* SB MO-injected embryos show a weaker band at 376 bp and a faint alternative band at 275 bp. Sequencing of the shorter band demonstrated the expected excision of exon 5 (data not shown). *eef1α1* is used as a housekeeping gene control indicating an equal expression of *eef1α1* in both samples. (B) Schematic of *plxna1b* gene, depicting the target sites of the translational blocking (TB MO) and the splice blocking Morpholinos (SB MO) (red). Note that the *plxna1b* SB MO and the *plxna1b* TB MO have been established before in a model for wound healing but not the early embryonic development.[18,19]

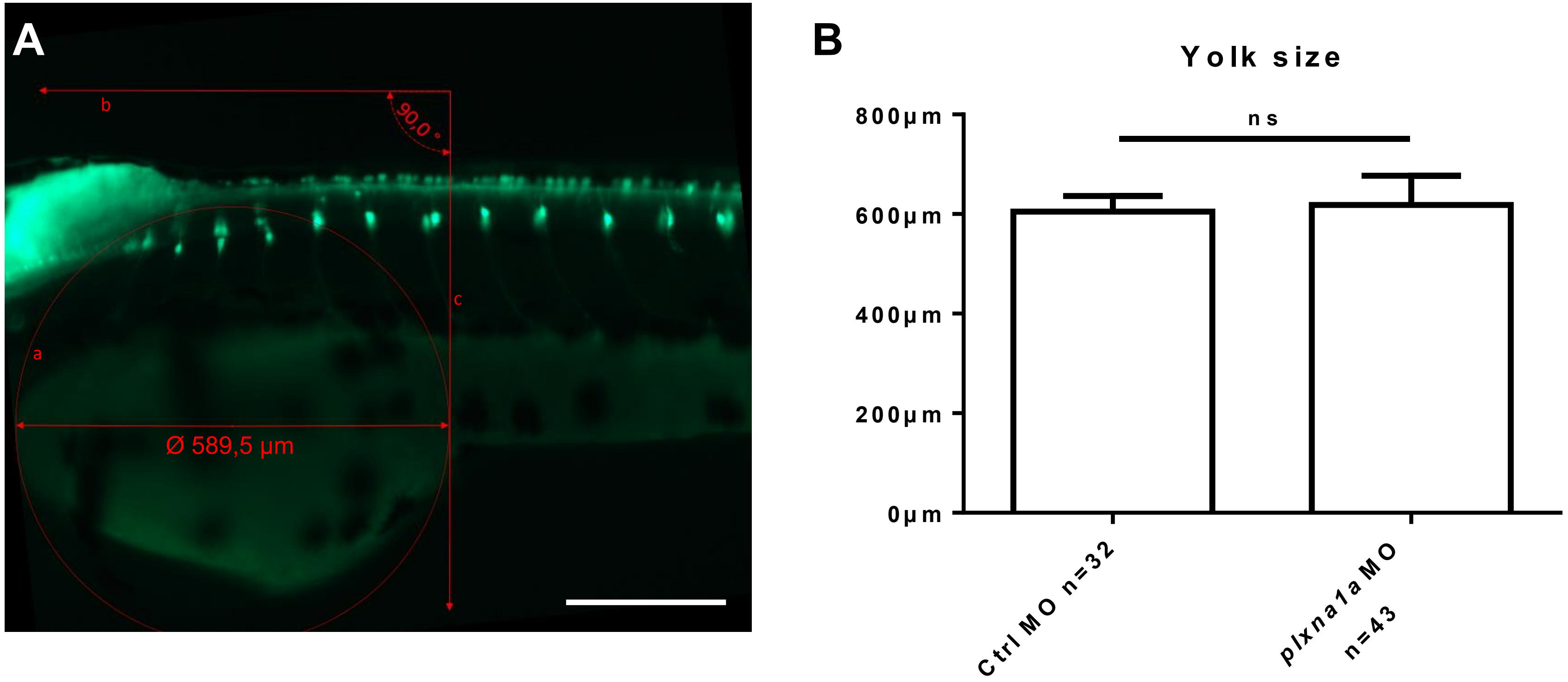
A
b
90,0 °
c
a
Ø 589,5 µm
B
Yolk size
800µm
600µm
400µm
200µm
0µm
ns
Ctrl MO n=32
plxna1a MO
n=43

Figure S6. Additional information on DRG counting.

(A) DRG counting was performed as visualized: The yolk was encircled (a) and the diameter was measured. Next, a line was drawn parallel to the spinal cord (b). From this line, a tangent towards the encircled yolk was drawn in 90° angle (c). For quantification of DRG, every DRG cranial from this tangent was counted.

(B) Yolk size comparison of Ctrl MO and *plxna1a* SB MO-injected fish. The diameter is not varying significantly (p-value 0,25; unpaired t-test, N=3). Scale bar 200µm. ns not significant.

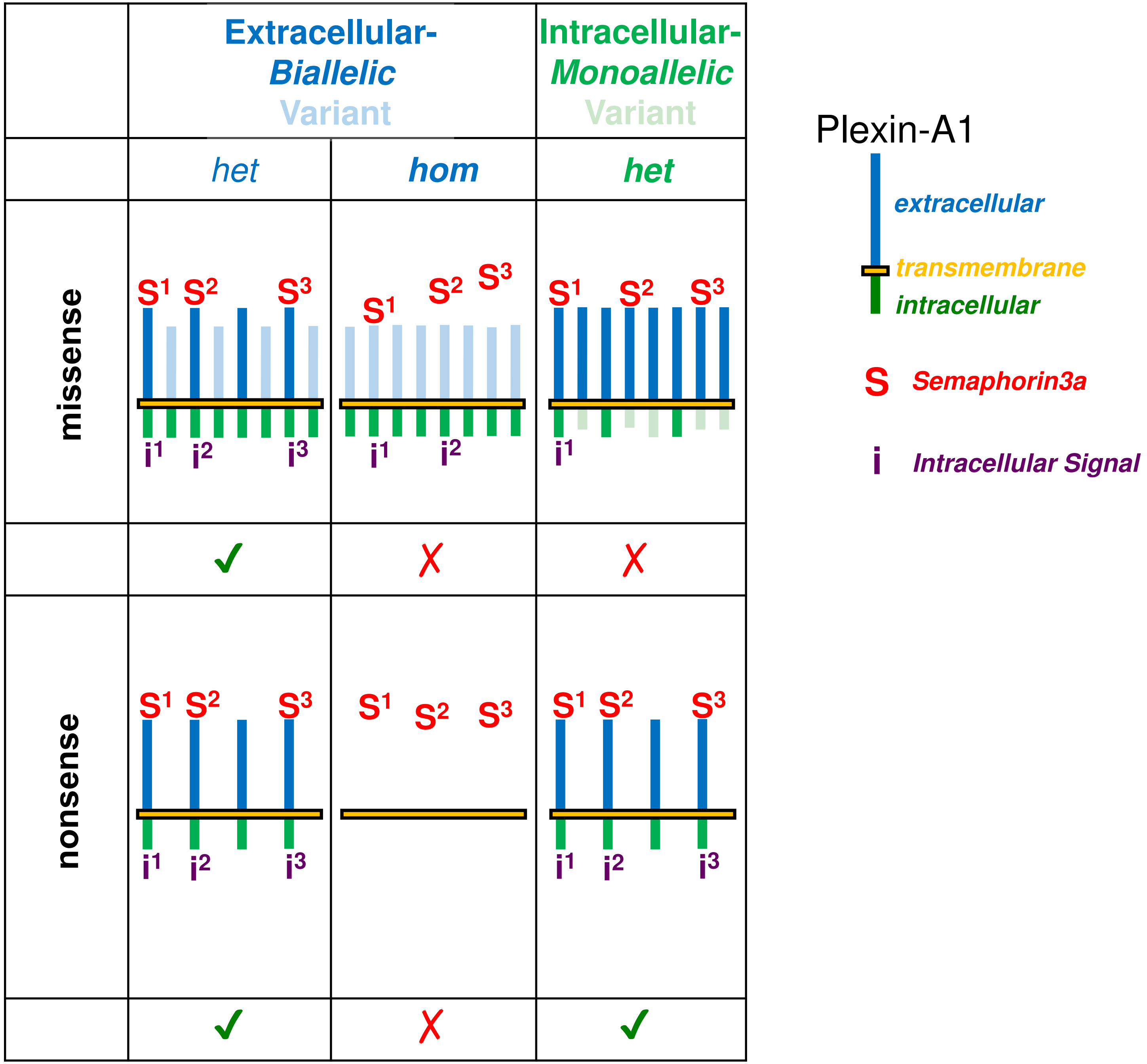

Extracellular-
Biallelic
Variant
Intracellular-
Monoallelic
Variant
het
hom
het
missense
nonsense
S1 S2 S3
i1 i2 i3
✓
✗
Plexin-A1
extracellular
transmembrane
intracellular
S Semaphorin3a
i Intracellular Signal

Figure S7. Hypothetic Plexin-A1 signaling model

A concept explaining the allelic heterogeneity at the *PLXNA1* locus leading to the disease phenotype via impaired transmembrane signaling as the common mechanism. Provided that extracellular signal (e.g. Semaphorin3a) is the rate-limiting factor of the Plexin-A1 signaling process over the membrane (orange) - extracellular localized variants (nonsense or missense, light-blue) can still convey proper signaling if present in a heterozygous state (left column). Only if the extracellular localized variant is in a homozygous state Plexin-A1 signaling would be disturbed (middle column) and the extracellular variant therefore only “effective”, if in a biallelic state. For intracellular localized variants a proper signaling is not preserved if the variant is present heterozygously, because of interference within the signaling cascade (right column). Therefore, intracellular variants are potentially already “effective” in a monoallelic state. This may represent a possible dominant negative effect – a missense variant in the intracellular domains may poison the dimerized Plexin-A1 receptor macromolecule.

Legend

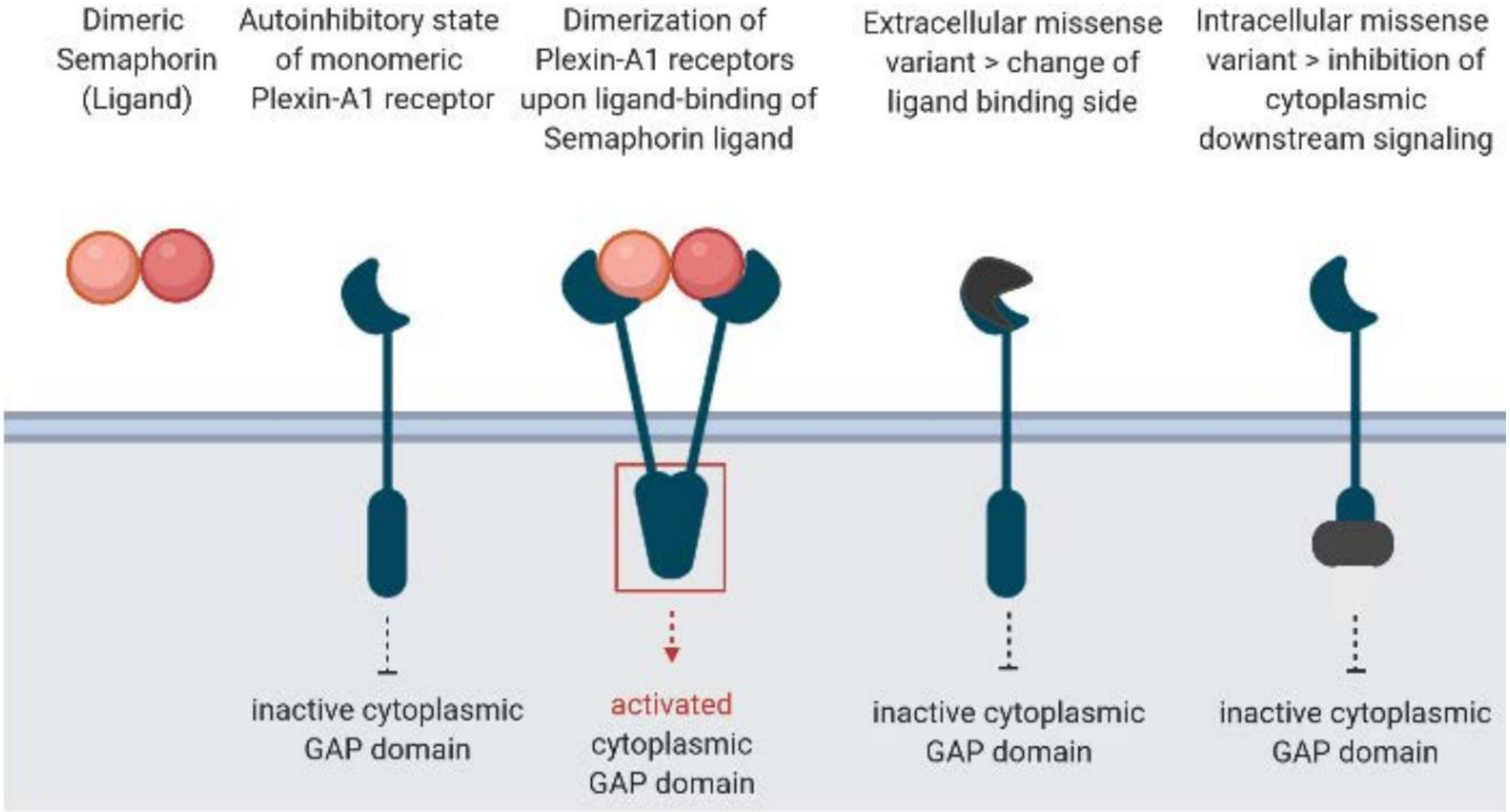

Loss of function (LoF) variants result in „nonsense mediated decay (NMD)“ irrespective of their extracellular or intracellular localization. Hereof excluded might be LoF variants that reside in the last exon of *PLXNA1*, experiencing "NMD escape". Hence, monoallelic heterozygotes of either extracellular or intracellular LoF variants should not experience impaired Plexin-A1 signaling. All expressed Plexin-A1 proteins bind with Semaphorins and initiate signaling. On the contrary, individuals with biallelic LoF variants do not express any Plexin-A1 protein.

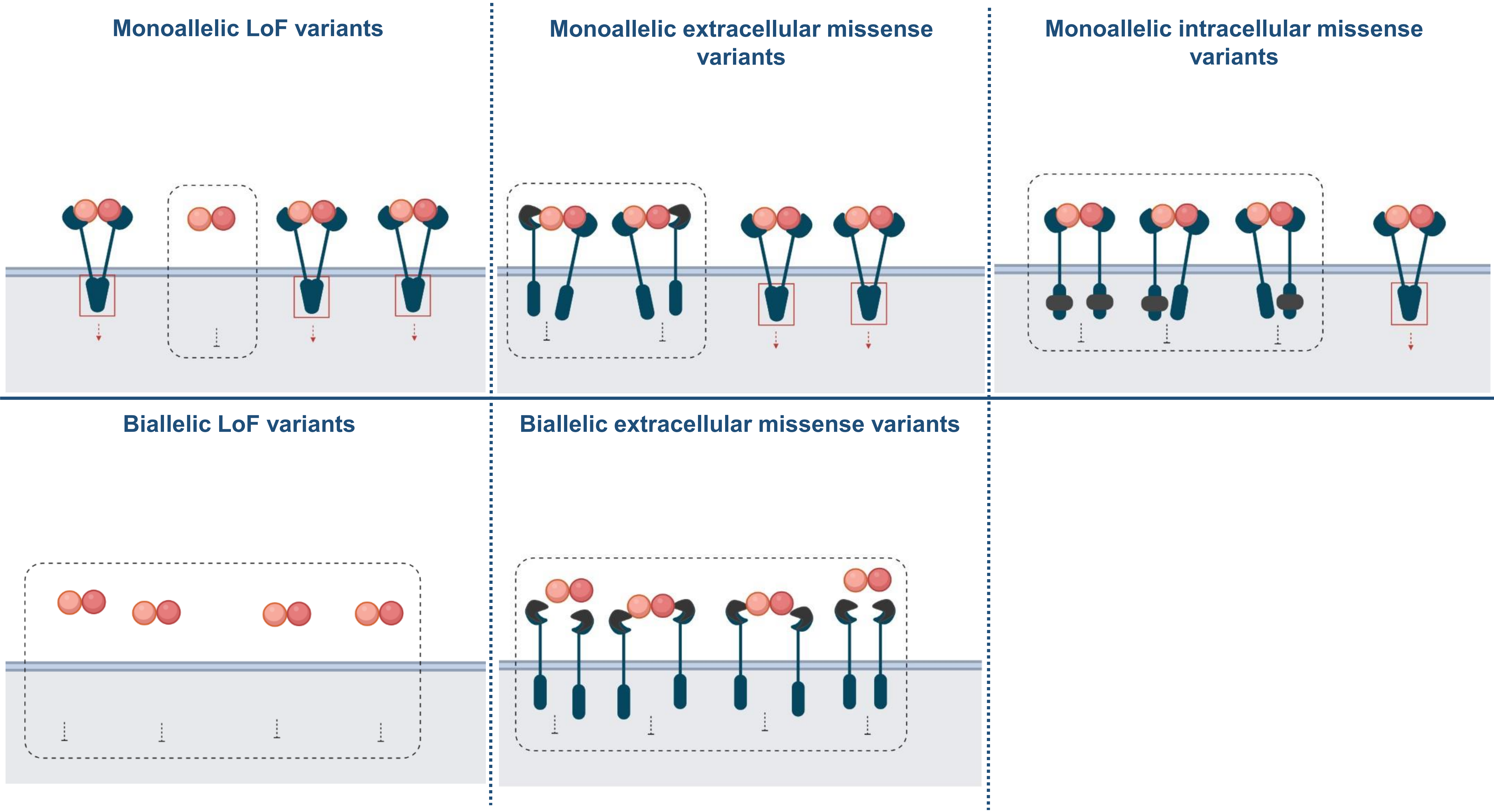
Monoallelic LoF variants
Monoallelic extracellular missense variants
Monoallelic intracellular missense variants
Biallelic LoF variants
Biallelic extracellular missense variants

Figure S8. Hypothetic Plexin-A1 cellular effects on signaling

Supplementary References

1. Vetro A, Pisano T, Chiaro S, et al. Early infantile epileptic-dyskinetic encephalopathy due to biallelic PIGP mutations. *Neurol Genet*. 2020;6(1). doi:10.1212/NXG.0000000000000387
2. Tanaka AJ, Cho MT, Millan F, et al. Mutations in SPATA5 Are Associated with Microcephaly, Intellectual Disability, Seizures, and Hearing Loss. *Am J Hum Genet*. 2015;97(3):457-464. doi:10.1016/j.ajhg.2015.07.014
3. White JJ, Mazzeu JF, Coban-Akdemir Z, et al. WNT Signaling Perturbations Underlie the Genetic Heterogeneity of Robinow Syndrome. *Am J Hum Genet*. 2018;102(1):27-43. doi:10.1016/j.ajhg.2017.10.002
4. Cummings BB, Marshall JL, Tukiainen T, et al. Improving genetic diagnosis in Mendelian disease with transcriptome sequencing. *Sci Transl Med*. 2017;9(386). doi:10.1126/scitranslmed.aal5209
5. Aitken S, Firth H V, McRae J, et al. Finding Diagnostically Useful Patterns in Quantitative Phenotypic Data. *Am J Hum Genet*. 2019;105(5):933-946. doi:10.1016/j.ajhg.2019.09.015
6. Weitensteiner V, Zhang R, Bungenberg J, et al. Exome sequencing in syndromic brain malformations identifies novel mutations in ACTB, and SLC9A6, and suggests BAZ1A as a new candidate gene. *Birth Defects Res*. 2018;110(7):587-597. doi:10.1002/bdr2.1200
7. Coban-Akdemir Z, White JJ, Song X, et al. Identifying Genes Whose Mutant Transcripts Cause Dominant Disease Traits by Potential Gain-of-Function Alleles. *Am J Hum Genet*. 2018;103(2):171-187. doi:10.1016/j.ajhg.2018.06.009
8. Dias CM, Punetha J, Zheng C, et al. Homozygous Missense Variants in NTNG2, Encoding a Presynaptic Netrin-G2 Adhesion Protein, Lead to a Distinct Neurodevelopmental Disorder. *Am J Hum Genet*. 2019;105(5):1048-1056. doi:10.1016/j.ajhg.2019.09.025
9. Monies D, Abouelhoda M, Assoum M, et al. Lessons Learned from Large-Scale, First-Tier Clinical Exome Sequencing in a Highly Consanguineous Population. *Am J Hum Genet*. 2019;104(6):1182-1201. doi:10.1016/j.ajhg.2019.04.011
10. Mencacci NE, Kamsteeg EJ, Nakashima K, et al. De Novo Mutations in PDE10A Cause Childhood-Onset Chorea with Bilateral Striatal Lesions. *Am J Hum Genet*. 2016;98(4):763-771. doi:10.1016/j.ajhg.2016.02.015
11. Willems PJ, Gatti R, Darby JK, et al. Fucosidosis revisited: a review of 77 patients. *Am J Med Genet*. 1991;38(1):111-131. doi:10.1002/ajmg.1320380125
12. Ediz SS, Aralasmak A, Yilmaz TF, Toprak H, Yesil G, Alkan A. MRI and MRS findings in fucosidosis; a rare lysosomal storage disease. *Brain Dev*. 2016;38(4):435-438. doi:10.1016/j.braindev.2015.09.013
13. Posey JE, Harel T, Liu P, et al. Resolution of Disease Phenotypes Resulting from Multilocus Genomic Variation. *N Engl J Med*. 2017;376(1):21-31. doi:10.1056/NEJMoa1516767
14. Kosfeld A, Kreuzer M, Daniel C, et al. Whole-exome sequencing identifies mutations of TBC1D1 encoding a Rab-GTPase-activating protein in patients with congenital anomalies of the kidneys and urinary tract (CAKUT). *Hum Genet*. 2016;135(1):69-87. doi:10.1007/s00439-015-1610-1
15. Demos M, Guella I, DeGuzman C, et al. Diagnostic Yield and Treatment Impact of Targeted Exome Sequencing in Early-Onset Epilepsy. *Front Neurol*. 2019;10:434. doi:10.3389/fneur.2019.00434
16. St. Clair RM, Emerson SE, D’Elia KP, et al. Fyn-dependent phosphorylation of PlexinA1 and PlexinA2 at conserved tyrosines is essential for zebrafish eye development. *FEBS J*. 2018;285(1):72-86. doi:10.1111/febs.14313

17. Roy A, Kucukural A, Zhang Y. I-TASSER: A unified platform for automated protein structure and function prediction. *Nat Protoc*. 2010;5(4):725-738. doi:10.1038/nprot.2010.5
18. Ton Q V., Kathryn Iovine M. Semaphorin3d mediates Cx43-dependent phenotypes during fin regeneration. *Dev Biol*. 2012;366(2):195-203. doi:10.1016/j.ydbio.2012.03.020
19. Yoo SK, Pascoe HG, Pereira T, et al. Plexins function in epithelial repair in both Drosophila and zebrafish. *Nat Commun*. 2016;7. doi:10.1038/ncomms12282
